# Synthesis and in Silico Modelling of the Potential Dual Mechanistic Activity of Small Cationic Peptides Potentiating the Antibiotic Novobiocin against Susceptible and Multi-Drug Resistant *Escherichia coli*

**DOI:** 10.3390/ijms21239134

**Published:** 2020-11-30

**Authors:** Ilaria Passarini, Pedro Ernesto de Resende, Sarah Soares, Tadeh Tahmasi, Paul Stapleton, John Malkinson, Mire Zloh, Sharon Rossiter

**Affiliations:** 1 School of Life and Medical Sciences, University of Hertfordshire, College Lane, Hatfield AL10 9AB, UK; i.passarini@herts.ac.uk (I.P.); tadehtahmasi@gmail.com (T.T.); 2 UCL School of Pharmacy, 29-39 Brunswick Square, London WC1N 1AX, UK; pedroderesende@gmail.com (P.E.d.R.); sarahapso@gmail.com (S.S.); p.stapleton@ucl.ac.uk (P.S.); j.malkinson@ucl.ac.uk (J.M.); 3 NanoPuzzle Medicines Design, Stevenage SG1 2DX, UK

**Keywords:** antimicrobial peptides, antimicrobial resistance, efflux pump inhibitor, molecular similarity, molecular dynamics, docking, peptide synthesis, antibiotic potentiation

## Abstract

Cationic antimicrobial peptides have attracted interest, both as antimicrobial agents and for their ability to increase cell permeability to potentiate other antibiotics. However, toxicity to mammalian cells and complexity have hindered development for clinical use. We present the design and synthesis of very short cationic peptides (3–9 residues) with potential dual bacterial membrane permeation and efflux pump inhibition functionality. Peptides were designed based upon in silico similarity to known active peptides and efflux pump inhibitors. A number of these peptides potentiate the activity of the antibiotic novobiocin against susceptible *Escherichia coli* and restore antibiotic activity against a multi-drug resistant *E. coli* strain, despite having minimal or no intrinsic antimicrobial activity. Molecular modelling studies, via docking studies and short molecular dynamics simulations, indicate two potential mechanisms of potentiating activity; increasing antibiotic cell permeation via complexation with novobiocin to enable self-promoted uptake, and binding the *E. coli* RND efflux pump. These peptides demonstrate potential for restoring the activity of hydrophobic drugs.

## 1. Introduction

Antibiotic resistance is a growing global emergency, with few novel antibiotics on the horizon. Multi-drug resistant Gram-negative bacteria in particular are a progressively more concerning threat, with many drug classes suffering from poor cell penetration. Restoring the potency of antibiotics that are no longer in clinical use due to lack of efficacy or bacterial resistance is one potential route to extend the life of current therapies. Cationic antimicrobial peptides (AMPs) are found widely in nature and have been of interest as novel therapeutics [1,2,3]. AMPs are believed to act via interaction with bacterial membranes, leading to increased cell permeability or membrane disruption. There is a need to develop novel peptides or peptidomimetics, which maintain these desirable properties but avoid the structural complexity and toxicity that have hampered clinical development to date.

Around 5000 AMPs have been reported, with ca. 1000 having a published structure [1]. Our previous investigation of the sequences and conformations of a library of cationic antimicrobial peptide structures highlighted the importance of alternating basic and aromatic amino acid residues [1]. This is thought to be important in allowing the peptides to assume the amphipathic conformation that is required for them to interact with the bacterial membrane. Additionally, the pairing of basic and aromatic residues could mimic the structure of the RND efflux pump inhibitor phenylalanine-arginine-β-naphthylamide (PAβN), which follows a similar aromatic-basic-aromatic motif [4]. This led to the hypothesis that small peptides with such alternating amino acid sequences could potentially retain antimicrobial activity whilst also overcoming a major resistance mechanism. Naturally occurring AMPs are usually between 11 and 50 residues long [5]. It has been reported that the antimicrobial pharmacophore of natural peptides requires a minimum of 5–6 residues [6]; although shorter antimicrobial peptides bearing unnatural amino acids are reported [7,8]. With these features in mind, a series of short peptides was designed to test our hypothesis.

## 2. Results and Discussion

### 2.1. Design and Synthesis of Peptides

A series of peptides was designed to incorporate the residues required for amphipathic conformation and potential efflux pump binding. It was decided to limit the length of the designed peptides to a maximum of nine amino acids with the intention of reducing toxicity. The aim was to combine the antimicrobial and cell permeation-enhancing properties of naturally occurring AMPs with efflux pump inhibition exhibited by PAβN, for potential activity either alone or in combination with other therapeutic agents. Therefore, peptides from 3–5 residues were included, as, in our models, even the shortest sequences still exhibited the amphipathic conformations required for cell membrane interaction, and more closely resembled PAβN. Tryptophan and phenylalanine were selected as hydrophobic aromatic amino acids. Additionally, a subset of peptides included a number of proline residues, based on the indication that proline-rich peptides (ProAMPs) are also linked to Gram-negative antimicrobial activity with low toxicity towards mammalian cells [7,8,9,10]. These peptides were designed by rationally modifying the highly preserved ProAMP sequence PPYLPRPRPPR [10]. Twenty-seven peptides in total were investigated; these were synthesised in-house by solid phase methods (Figures S1–S26, Tables S1–S3) or obtained from commercial suppliers. All peptides were amidated at the C-terminus, in order to prevent the formation of a negative charge, which would affect the ability of the peptide to bind the lipopolysaccharide on the Gram-negative envelope.

### 2.2. Antimicrobial and Potentiating Properties

The Minimum Inhibitory Concentrations (MIC) of compounds **1**–**27** were evaluated through a broth microdilution assay against susceptible strains of *Escherichia coli* (Gram–negative) and *Staphylococcus aureus* (Gram-positive). None of the peptides exhibited significant intrinsic antimicrobial activity (MIC values 64 μg/mL for compound **6**, >128 μg/mL for all others, Table S4), indicating that, although predicted to interact with the lipopolysaccharide (LPS) in the outer membrane of the Gram-negative species, they were not able to disrupt bacterial membranes in either species sufficiently to cause an antibacterial effect on their own [9].

The peptides were also tested in combination with antibiotics novobiocin and norfloxacin. Novobiocin has low efficacy against Gram-negative bacteria due to its hydrophobicity, giving limited cell permeability [11]. Thirteen of the peptides exhibited some potentiation of novobiocin activity against susceptible bacteria, with five of the peptides exhibiting a strong potentiating effect against both susceptible (10418) and multi-drug resistant (G69) strains of the Gram-negative bacterium *E. coli* (Table 1) [12]. Two of the peptides, **6** and **27**, were able to elicit a 256-fold reduction in the MIC of novobiocin against the susceptible strain—an indication that the peptides may be assisting permeation of the LPS-containing membrane barrier.

Most excitingly, pentapeptides **6**, **10**, **15**, **19** and proline-rich nonapeptide **27** were also found to restore the susceptibility of the multi-drug resistant *E. coli* to novobiocin. Weaker potentiation effects were also observed for a number of other peptides, from 3–8 residues in length, in the set, including significant activity of tetrapeptides **5** and **9** against both bacterial strains. This represents a potentially significant advance, as these small peptides may have potential to rescue “lost” antibiotics by restoring their activity to a clinically useful level against multi-drug resistant bacteria (Table 1).

In order to gather more evidence on the potential mechanisms of action, the effects of the peptides in combination with the quinolone antibiotic norfloxacin were determined. Norfloxacin is active against Gram-negative bacteria, generally achieving entry via porin channels, but is also able to enter the bacterial cell via the membrane in porin-deficient mutants [13]. Therefore, any membrane permeability mechanism would not be expected to have a significant effect on improving the activity of norfloxacin in porin-competent strains. This is indeed what was observed (Table S5), which added weight to our hypothesis that our very short peptides may be acting as membrane permeability enhancers for novobiocin.

We hypothesized that the peptides could act via the formation of a novobiocin-peptide complex, capable of better penetrating the bacterial membrane, by a mechanism similar to self-promoted uptake. This could lead to the increased activity of novobiocin observed against the susceptible *E. coli* strain (Table 1), in agreement with previous literature studies [10,11,14,15].

### 2.3. In Silico Studies

To further explore this assumption, interactions between that peptides 6 and 27 could establish with antibiotics novobiocin and norfloxacin were investigated in silico. The conformations of the two antibiotics in explicit solvent were initially obtained by submitting two systems containing the peptide and either novobiocin or norfloxacin to simulated annealing and molecular dynamics (MD) simulation experiments.

The 50 ns MD simulations of novobiocin with both compound **27** and compound **6** resulted in a trajectory with complex formation involving all molecules of the system. In the case of compound **27**, all molecules were involved in a single major complex, whereas for compound **6**, all molecules were incorporated within two separate complexes (Figure S27). For norfloxacin, the 50 ns MD simulation resulted in little or no complex formation. The simulated trajectory with compound **27** resulted in no complex formation. The simulation with compound **6** resulted in limited complex formation; a single complex of two molecules of norfloxacin and one molecule of peptide was observed, however, the remaining three norfloxacin molecules remained scattered throughout the system (Figure S28).

The surfaces areas of the structures observed were also generated and analyzed, using Vega ZZ version 3.2.0 to explore a possible role of complex formation in the enhancement of drug permeability [16]. Molecular lipophilicity potential (MLP), with a probe radius of 1.5 and mesh size of 0.60, Broto log P, lipole (a measure of the lipophilicity distribution) and virtual log P were also obtained (Table 2).

**Figure 1:**
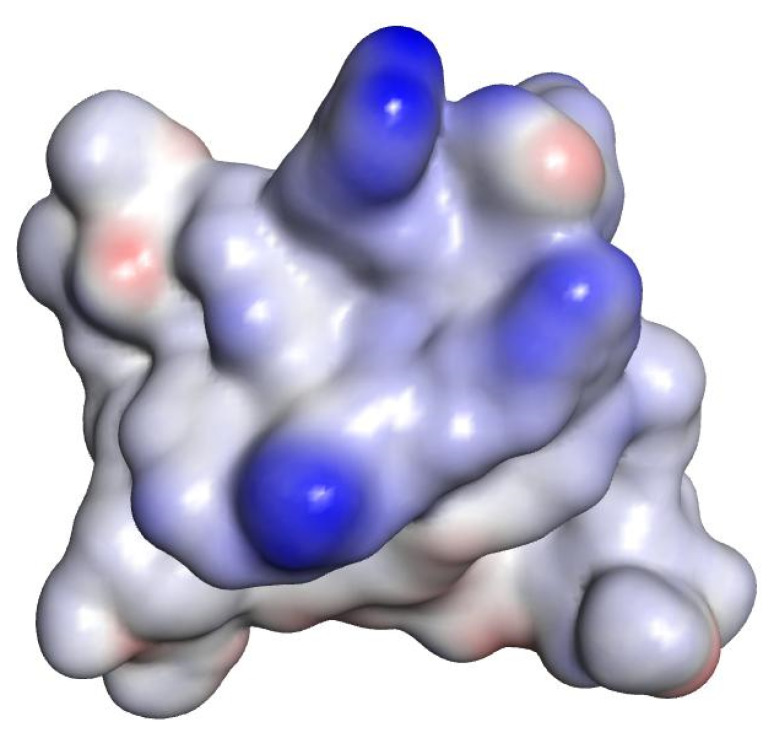
Molecular electrostatic potential (MEP) of one complex formed by one molecule of compound **6** (peptide sequence WRWRW-NH_2_) and three novobiocin molecules. The hydrophilic positively charged side chains (blue) are protruding from the complex. Hydrophobic regions are shown in grey and negative dipoles in red.

**Figure 2:**
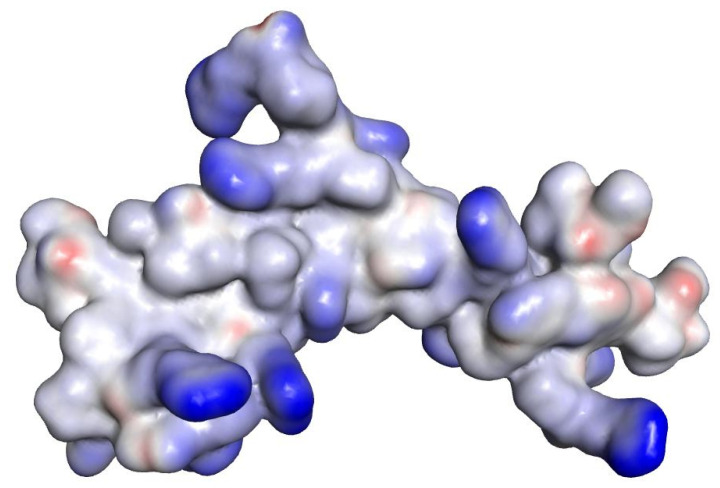
MEP of the representative complex formed by compound **27** (peptide sequence RPRRPRLPW-NH_2_) and novobiocin molecules. Positive values are shown in blue, hydrophobic regions in grey and negative values in red.

**Figure 3:**
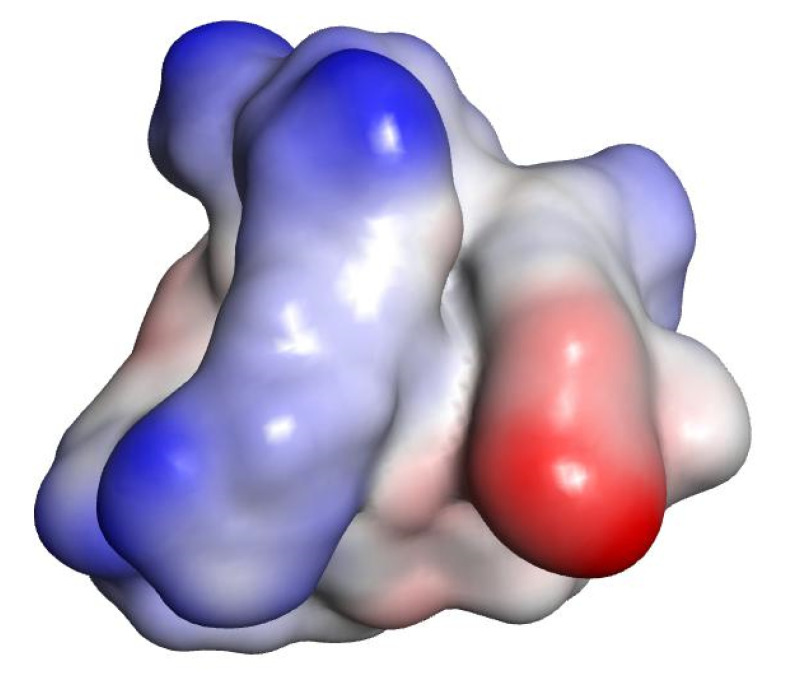
MEP of the representative complex formed by one compound **6** (WRWRW-NH_2_) and three norfloxacin molecules. Negative values are shown in red, hydrophobic regions in grey and positive values in blue.

Polar surface areas and molecular lipophilicity potential (MLP) maps were also generated with VegaZZ. The molecular electrostatic potential (MEP) maps were also generated using DS visualizer v 4.0 (Figure 1, Figure 2 and Figure 3).

**Figure 4:**
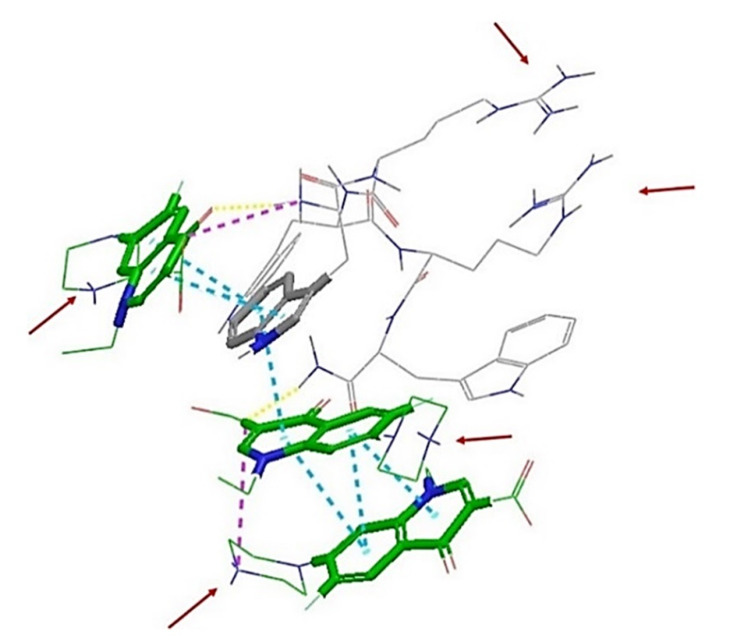
Intermolecular interactions observed in the complex formed between norfloxacin and peptide **6** (WRWRW-NH_2_) at the end of 50 ns molecular dynamics (MD) simulation. A conjugation system formed by a series of π-stacking interactions (blue) is formed between the quinolone rings of three norfloxacin molecules and the indole ring of Trp1 of the peptide. The complex is further stabilized by salt bridges (purple) and hydrogen bonds (yellow). The positively-charged side chains of the arginine residues extend out from the complex; this, along with the protonated piperazine rings (positive charges are indicated by the red arrows), confers a hydrophilic character to the complex, possibly preventing this complex from entering through self-promoted uptake.

**Figure 5:**
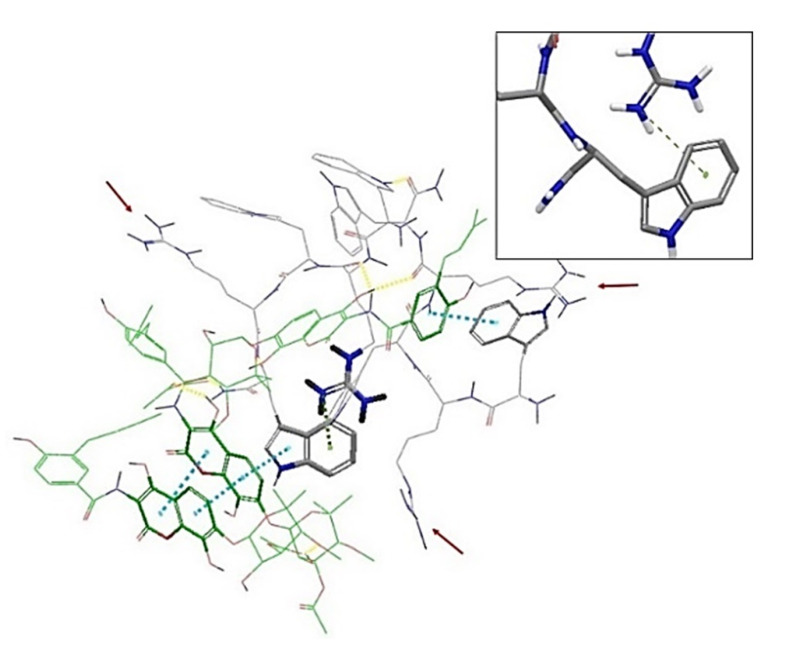
Intermolecular interactions observed in the complex formed between novobiocin and peptide **6** (WRWRW-NH_2_) at the end of 50 ns MD simulation. The complex is held together by a series of π-stacking interactions (blue) and hydrogen bonds (yellow). Cationic side chains are also protruding from the complex; however, Arg2 is shielded by a π–cation interaction (green, inset) with the indole ring of Trp5. This, combined with the absence of charges on the novobiocin possibly results in better amphipathic character, enhancing the uptake of novobiocin through the bacterial membrane.

The interactions between peptide and antibiotic molecules were analyzed (Figure 4 and Figure 5). It was observed that the formation of antibiotic-peptide complexes occurs with both antibiotics. In particular, for the complex observed between norfloxacin and compound **6**, the norfloxacin quinolone rings form π-stacking interactions with themselves and with the indole ring of Trp1 and Trp3; two norfloxacin carboxylic acids form H-bonds with Trp3, Arg 4 and Trp5; a salt bridge can also be observed between the charged carboxylic acid of one norfloxacin and Arg4 (Figure 4).

For the complex formed between three novobiocin and two peptide molecules, six hydrogen bonds are observed with Arg1 of one peptide and Arg5 of the other, whereas the hydroxyl substituent of one novobiocin coumarin ring established H-bonding with Arg4 of one peptide and Arg2 of the other. π-Stacking is observed between the aromatic rings of novobiocin and Trp1 of one peptide and Trp5 of the other; and intramolecular π-cation interaction is observed between Arg2 and Trp5 of the second peptide, possibly shielding a positive charge (Figure 5).

The molecular lipophilicity potentials (MLP) of two of the largest complexes observed were generated. Molecular properties including surface area and virtual logP were investigated as a measure of the conformation-dependent hydrophobicity. For all complexes, it was observed that surface areas increased 2.4 to 3.1-fold in comparison to that obtained for one free molecule of antibiotic, whereas virtual logP values decreased in all cases, indicating that the complexes formed are more hydrophilic than the corresponding free antibiotics.

**Figure 6:**
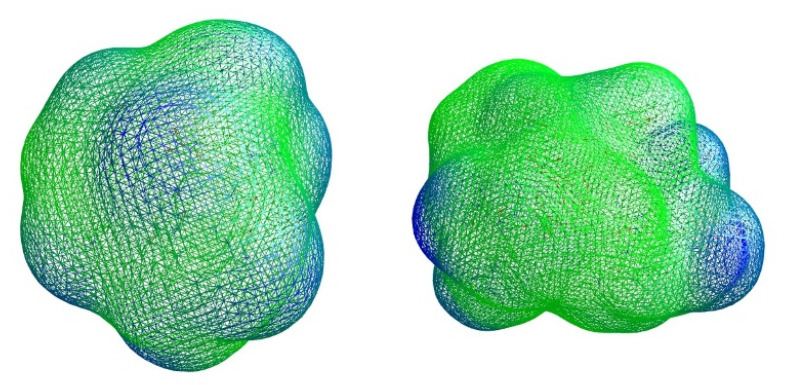
Dashed molecular lipophilicity potential (MLP) maps obtained for a complex formed by three molecules of norfloxacin and one of peptide **6** (**left**) and a complex formed by three molecules of novobiocin and two of peptide **6** (**right**). Hydrophobic regions are colored in green and hydrophilic regions in blue.

In the case of norfloxacin, the situation shifts from a small molecule—which is able to penetrate the Gram-negative envelope—to a very large complex. This allows for a rather distinct segregation of charges and a strong hydrophilic character with an overall amphipathic surface. Additionally, this complex displays several patches of negative charge, which are likely to prevent it from undergoing the self-promoted uptake pathway (Figure 3 and Figure 6 left). Conversely, in the case of novobiocin, the scenario moves from a hydrophobic molecule to a large complex with a distinct amphipathic character, where the positive charges are neatly segregated to one portion of the structure (Figure 1, Figure 2 and Figure 3 right). These characteristics could allow the complex to potentially benefit from the self-promoted uptake pathway, thus increasing the uptake of a molecule that would otherwise not possess the physicochemical properties required to bypass the LPS and bacterial envelope to reach the intracellular drug target [16].

**Figure 7:**
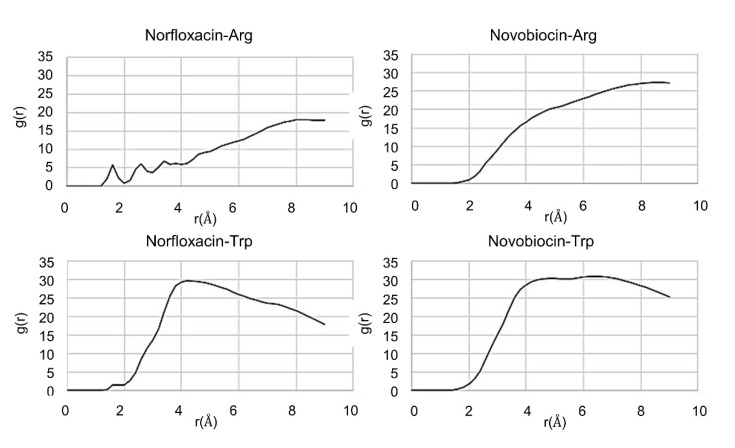
Plots representing the radial distribution function (RDF) patterns obtained from the trajectory of 50 ns MD simulation of a system containing peptide 6 (WRWRW-NH_2_) and either novobiocin or norfloxacin.

In support of these observations, radial distribution functions (RDF) were generated from the trajectories, by looking at the probability of finding the antibiotic at a certain distance from either an arginine or tryptophan residue present in the system throughout the simulation (Figure 7).

Norfloxacin is characterized by the formation of hydrogen bonds with tryptophan and arginine, represented by the peaks observed in the 1.5–2.5 Å region. Conversely, no hydrogen bond formation with the peptide residues is clearly observed for novobiocin. The π–cation interactions and π–π stacking interactions, which occur at a maximum distance of 6.6 Å and 4.4 Å, respectively, appear more favorable for novobiocin than for norfloxacin. Finally, all residues, and in particular arginine, appear more likely to spend more time in close proximity to novobiocin than norfloxacin. This suggests that compound **6** is less likely to form persistent complexes with norfloxacin than novobiocin, which seems to support our hypothesis of the role of complex formation in increasing passive uptake.

A second hypothesis was based on the potentiation results obtained for the RND efflux pump inhibitor PAβN (Table 1). These show that PAβN is able to restore the activity of novobiocin towards the resistant strain *E. coli* G69, with MIC values similar to those observed in the presence of the peptides. PAβN, which has been reported to increase cell permeability in *Pseudomonas aeruginosa* [17], also has a small effect for the susceptible *E. coli* 10418: a twofold reduction in MIC for novobiocin is seen at a PAβN concentration of 72 μM (32 μg/mL) [18,19]. However, PAβN is not able to affect the MIC of norfloxacin against susceptible or resistant *E. coli*, even at a higher concentration of 0.14 mM (64 μg/mL). The exact mechanisms of resistance of *E. coli* G69 are not fully elucidated, but norfloxacin resistance is likely to be due to target site genetic mutations in the gyrA/parC genes, therefore activity cannot be restored by prevention of drug efflux. Compounds **6** and **27** show a similar pattern to PAβN against the multi-drug resistant *E. coli* G69, indicating they could also behave as efflux pump inhibitors. Interestingly, peptides **6** and **27** can provide this synergism at concentrations of 36 μM and 27 μM, respectively, lower than that observed for PAβN (72 μM).

**Figure 8:**
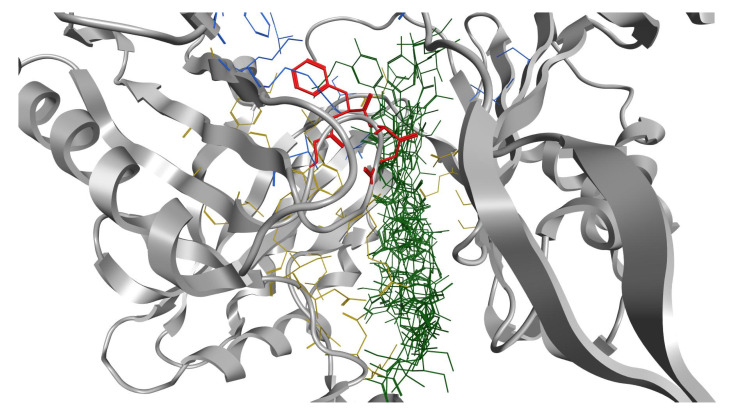
All 15 poses obtained for novobiocin (green) are located in the distal pocket (yellow). The proximal pocket is shown in blue and the loop in red.

**Figure 9:**
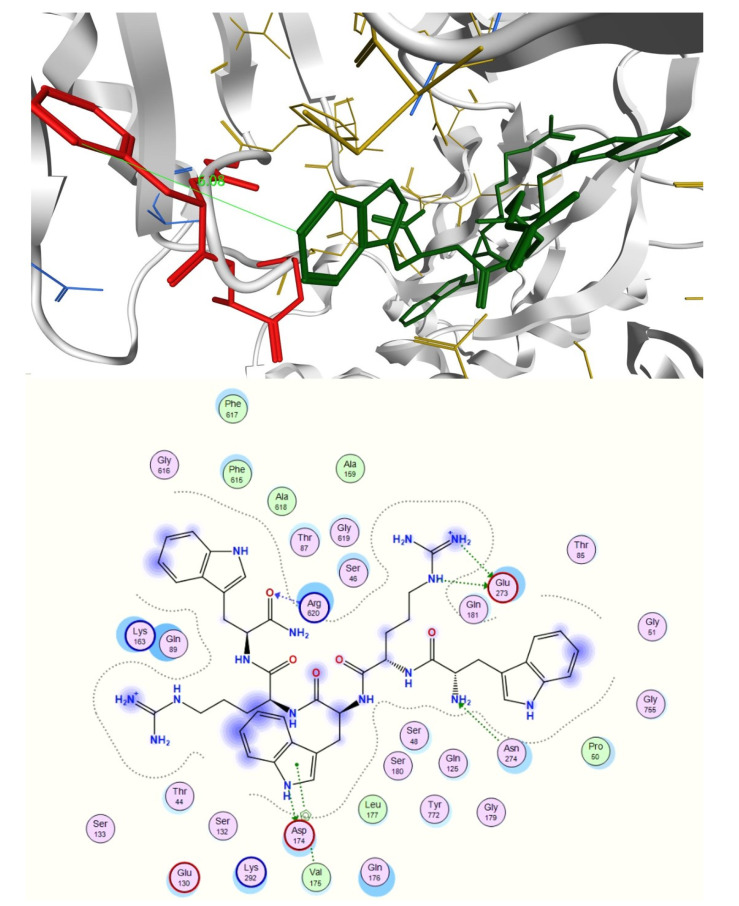
The most favorable pose of potentiating peptide **6**, WRWRW-NH_2_ (green) located in the distal binding pocket (yellow). The peptide interacts strongly with the loop (red) and in particular Trp6 forms a hydrophobic interaction with Phe617. The proximal binding pocket is shown in blue.

**Figure 10:**
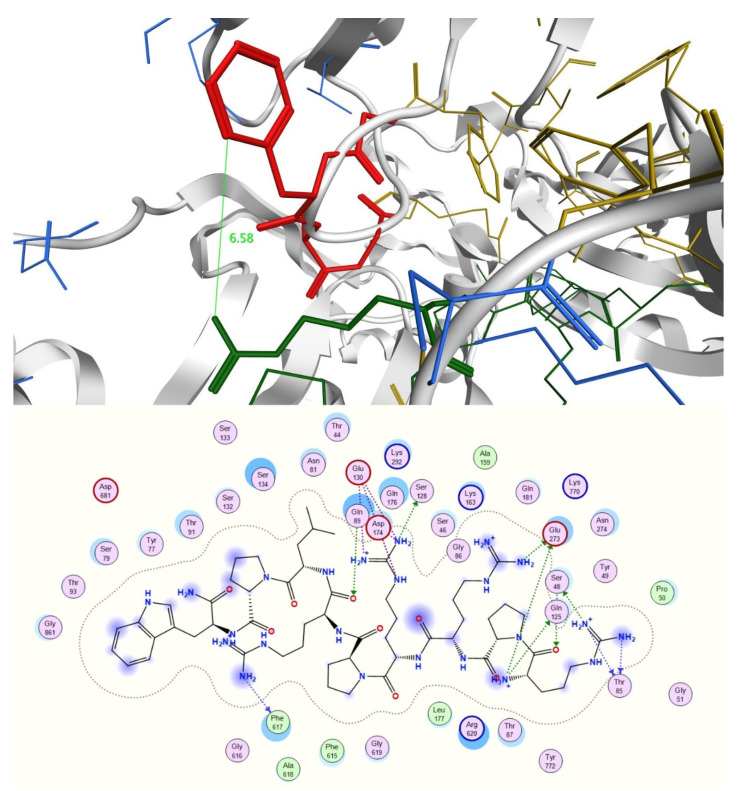
The most favorable pose of potentiating peptide **27**, RPRRPRLPW-NH_2_ (green) establishes interactions with both the distal (yellow) and proximal (blue) binding pocket. Interactions can be observed with all four residues forming the loop (red) and in particular Arg6 (bold green) interacts with Phe617.

To validate this theory, a blind docking study was performed. Norfloxacin, novobiocin, PAβN, the two most active peptides **6** and **27**, and a weakly-potentiating peptide from the group, peptide **3**, were docked into the periplasmic domain of the AcrB portion of the *E. coli* RND efflux pump, which was prepared from the PDB ID 4DX5 as previously described in the literature [20]. There is evidence that whilst the majority of RND substrates bind into a distal binding pocket, inhibitors such as PAβN also partially bind and reduce the flexibility of a loop that facilitates the movement and extrusion of substrates [20,21,22]. Interestingly, docking results showed that novobiocin and compounds **6** and **27** all bind to the distal binding pocket, whereas norfloxacin and PAβN favor the proximal pocket (Supplementary Materials, Figures S29–S34). Most interestingly, however, PAβN, compound **6** and compound **27** are found to strongly interact with the loop region (Figure 8, Figure 9 and Figure 10) and with high affinity (Table 3). Conversely, weakly potentiating compounds **3** and **23** were only seen to bind into the distal pocket with lower affinity and with no loop interaction. (Supplementary Materials, Figures S35–S38).

This lends weight to the hypothesis that the active compounds may also have the ability to inhibit efflux pumps with a mode of action related to that of PaβN.

In conclusion, we were able to demonstrate that small cationic peptides of fewer than ten residues, without intrinsic antimicrobial activity, can dramatically improve the activity of novobiocin against susceptible *E. coli* 10418. In addition, the peptides enable restoration of novobiocin activity against a multi-drug resistant *E. coli* strain. This synergism can be attributed to improved cell permeability, potentially via the formation of amphipathic complexes between novobiocin and the peptides. The peptides also interact with the loop between the two binding pockets of a model of the *E. coli* RND efflux pump, suggesting the possibility of a dual mechanistic effect, via efflux pump inhibition.

The antibiotic potentiating peptides described herein, although at an early design and mechanistic investigation stage, demonstrate that small peptide structures of this type could be developed to enhance antibiotic therapies. There are a number of antimicrobial peptides in current clinical use; however, peptide therapies are generally limited to topical or intravenous administration, due to their inherent instability for oral administration. Similarly, peptides with potentiating activity and a favorable safety profile could be further developed towards an intravenous combination therapy. Alternatively, there is the potential to use the active structural scaffolds to design peptidomimetic compounds that maintain these antibiotic potentiating features and have the additional potential to be orally bioavailable.

This finding represents an exciting first step in designing simple, small peptides, peptidomimetics or drug conjugates to improve the efficacy of older antibiotics against both susceptible and multi-drug resistant Gram-negative bacteria—a strategy that may be crucial in the fight against antibiotic resistance.

## 3. Materials and Methods

### 3.1. Design of Proline-Rich Cationic Peptides

The design of the proline-rich AMPs (sequences **22**–**27**) was based on the following modifications to the parent structure PPYLPRPRPPR [5,23]:

- RPRPRPL (**22**): alternating Arg and Pro residues are maintained from the from parent structure. Peptide **22** did not show any significant potentiation of the MIC of novobiocin. This was not surprising as it does not contain any aromatic residues.
- RPWPPR (**23**): contains the last six residues of the parent structure but one Arg is substituted with a Trp to include aromaticity. Peptide **23** shows potentiation, but only against the susceptible strain.
- WKPLPPR (**24**): the aromatic residue is moved at the N terminal to better mimic PAβN. A leucine is also included to make the peptide slightly longer and include the chance of establishing interactions. It was also investigated whether substituting Lys for Arg could improve potentiation but no synergism was observed.
- FKPLPPH (**25**): the terminal residues were substituted with different amino acids but with the same electronic properties (Trp1 with Phe and Arg7 with Hys respectively). Potentiation was restored but again only against susceptible strain.
- The terminal portion of the parent structure was once again considered (RPPR) and this moiety repeated twice to increase length. Additionally, two Arg residues were substituted with Trp to include aromaticity, but not at the N terminus (peptide **24** did not show any improvement). This yielded peptide RPPWRPPW (**26**), however no potentiation was seen against the multidrug resistant strain.
- Pro2 and 3 were therefore separated with two Arg residues to add flexibility. Pro6 was also substituted with neutral leucine to further increase flexibility. Additionally, bulky Trp4 was eliminated to see if activity could be obtained with only one aromatic residue but without making the peptide too long. This led to RPPWRPPW (**27**), which shows an exciting profile.

### 3.2. Solid Phase Peptide Synthesis

All Fmoc-protected amino acids, Rink amide MBHA resin and HBTU were purchased from GyrosProtein Technologies Inc., Tucson USA. Solvents and other reagents were purchased from Fisher Scientific, Loughborough, UK. Peptides **20**, **21** and **25** were purchased from ThermoFisher, Paisley, UK with a guaranteed >98% purity and accompanying confirmatory analytical data.

An amount of 0.15 g (0.1 mmol according to resin load) of Rink amide MBHA resin was used as solid support. The amino acid vials were packed with 0.4 mmol of FMOC-protected amino acid and equimolar amount of activating agent HBTU each. All couplings were carried out using high purity *N*,*N*-dimethylformamide (DMF) for peptide synthesis. Then, 0.4 M *N*-methylmorpholine in DMF was used for the activation step and 20% solution of piperidine in DMF for deprotection. The resin-bound peptide was then washed with a 1:1 mixture of water/methanol and dried in a desiccator overnight. Cleavage of the peptide from the resin was obtained by gentle agitation in 3 mL of a standard cocktail solution of 95% trifluoroacetic acid, 2.5% deionized water and 2.5% triisopropylsilane for 3 h. The undissolved resin was filtered away, and the peptide solution concentrated under vacuum for half an hour. The crude peptides were then crystallized from ice-cold diethyl ether and freeze dried.

#### Purification and Characterization

The crude samples were analyzed through RP-HPLC to initially assess their purity. All analytical chromatograms were obtained on an Agilent Technologies 1260 Infinity instrument. A C18-2 5 μm, 250 × 4.6 mm TemesilTM HPLC column was used. A mixture of 0.02% *v/v* TFA in water (solvent A) and 0.016% *v/v* TFA in aqueous acetonitrile (solvent B) was used as mobile phase with the following gradient: 1% to 100% B over 20 min, maintain 100% B for 5′ and then 100% to 0% B over 7′. Flow rate was set at 1 mL/min and elution was monitored at 214 nm.

The crude samples were then dissolved in the minimum amount of solvent A and purified through semi-preparative RP-HPLC with the following gradient: 100% A over 5′, then 0% to 100% B over 35′. Flow rate was set at 10 mL/min and elution monitored at 214 and 254 nm. A Varian Prostar 500 instrument was used, fitted with a Varian Polaris C18-A 10 μm, 100 × 212 mm semi-preparative column.

Fractions collected were further analyzed through analytical RP-HPLC with the same method described previously (Figures S1–S24). Clean fractions were combined and freeze-dried. Molar % yield was finally calculated. Synthesized peptides were further characterized through mass spectrometry (LC-MS) (Table S1). Total ion chromatograms were obtained on a Varian Prostar triple-quad LC-MS with integrated ESI detector. All pure samples were initially dissolved in 0.02% *v/v* trifluoroacetic acid in water and were then eluted through a C18-2 5 μm, 250 × 4.6 mm TemesilTM HPLC column. A mixture of 0.1% *v/v* formic acid in water (solvent A) and 0.1% *v/v* formic acid in acetonitrile (solvent B) was used as mobile phase with the following gradient: 10% to 90% B over 20 min, maintain 90% B for 5 min and then back at 10% B over 7 min, with flow rate set at 1 mL/min. The mass of the main peak corresponding to the molecular ion was confirmed with a Varian triple-quad mass spectrometer integrated with an electrospray ionization (ESI) detector with voltage capillary set at 80,000 V. Full HPLC and LC-MS data are available in the Supplementary Materials (Figures S1–S24, Table S1). The molar percentage yields of peptides after purification are given in Table 4. Peptides **6** and **27** were selected for further investigation and were analyzed using NMR spectroscopy, to confirm the expected sequence and connectivity of the residues (Figures S25 and S26; Tables S2 and S3). 1H, NOESY and TOCSY NMR were acquired in a 10% *v/v* D_2_O solution in water on a Bruker Avance 500 MHz spectrometer.

### 3.3. Biological Assays

The antibacterial activity of the peptides and peptide/antibiotic combinations were tested by the broth microdilution assay according to [24]. *Escherichia coli* NCTC 10,418 and *Staphylococcus aureus* NCTC 12,981 were obtained from the National Collection of Type Cultures, UK. *Escherichia coli* G69 was a clinical isolate as previously described [12].

Briefly, all bacterial strains were cultured on nutrient agar (Sigma-Aldrich, Gillingham, UK) plates and incubated for 24 h at 37 °C prior to MIC determination. In addition, known quantities of each test sample were dissolved in DMSO and then diluted in ISB (Iso- Sensitest Broth, Oxoid, Basingstoke, UK) to give a range concentration of 128–0 μg/mL, unless stated differently. The DMSO concentrations employed in all experiments showed no inhibitory effect towards bacterial growth. Finally, the overnight cultures of each of the tested strains were suspended to an inoculum density of approximately 1.0 × 108 CFU/mL in phosphate buffered saline (PBS), consisting of 137 mM NaCl, 3 mM KCl, 8 mM Na_2_HPO_4_, and 15 mM KH_2_PO_4_ (Oxoid, Basingstoke, UK). The cell suspensions were standardized by adjusting the optical density to 0.1 at 600 nm (Thermo Scientific UV-Vis Spectrophotometer, Abingdon, UK) before being diluted 1:100 in ISB prior to inoculation

The assays were performed by microdilution using 96-well microtiter plates with a final inoculum of 5 × 105 CFU/mL and each sample was tested in duplicate in at least two independent experiments in order to confirm the reliability of the data. Results were determined by visual inspection of the wells and the presence of an evident opaque medium or white pellets were indicative of bacterial growth. The MIC values were recorded as the lowest concentration at which no bacterial growth was detected.

### 3.4. Computational Studies

#### 3.4.1. Clustering of MD Trajectories

The interactions between peptides **6** (WRWRW-NH_2_) or **27** (RPRRPRLPW-NH_2_) with each of the two antibiotics, novobiocin and norfloxacin, were investigated in silico. The aqueous conformations of the peptides and the antibiotics were predicted as described in the previous section.

Two 40 Å cubic systems were then created using the package Packmol and adapted a command script [25]. The four systems generated included four novobiocin and three WRWRW-NH_2_, five norfloxacin and two WRWRW-NH_2_, six novobiocin and three RPRRPRLPW-NH_2_ and eight norfloxacin and two RPRRPRLPW-NH_2_, respectively. These proportions were set to mimic the molar ratios found at a concentration of 128 μg/mL of both peptide and antibiotic, at which synergistic effects were seen for novobiocin but not for norfloxacin.

These systems were then imported into Maestro and the systems were prepared using the system builder tool, by adding an extra 5 Å SPC solvent model in all directions from the box already generated in Packmol and adding 0.15 M NaCl. The default minimization protocol was applied and simulated annealing was then performed using Desmond and the OPLS2005 force field, by heating to 10 K in 30 ps, to 100 K in 100 ps, to 300 K in 200 ps, to 1000 K in 300 ps, keeping at 100 K over a further 500 ps and cooling at 300 K in 1000 ps. Once again, default 9.0 Å was selected as cut off value for short range electrostatic interactions. The Berendsen thermostat was used to regulate the simulation temperature, with 1.0 ps relaxation time within the NVT ensemble [26]. RESPA integrator was used to integrate equations, with an inner and outer time steps of 1.0 and 3.0 fs, respectively [27]. The energies were recorded at every 5 ps, and the analysis was performed on trajectory coordinates.

The last frames of the simulated annealing were exported and submitted to 50 ns MD simulations using Desmond and the OPLS2005 force field and both simulations were performed in duplicate. Short-range electrostatic interactions were cut off at default 9.0 Å, the Nose–Hoover thermostat was applied to keep temperature constant at 300 K and the Martyna–Tobias–Klein barostat was used to control pressure within the NPT ensemble [28,29]. Once again, the equation of motion was integrated with the RESPA integrator, with an inner time step of 2.0 fs and an outer time step of 6.0 fs [27]. The trajectories were saved at 5 ps intervals for analysis.

The trajectories obtained from the molecular dynamics simulations were then submitted to clustering using the Desmond trajectory clustering tool, available in Maestro version 11.8.012 (Schrödinger, New York, NY, USA) [30,31]. RMSD matrix was used as the metric for similarity calculations. Residues arginine and tryptophan as well as the antibiotic molecules were selected in the ASL panel and the RMSD was calculated at every 10th frame. Hierarchical clustering with average linkage was selected as clustering method and the five most populated clusters were saved as out.cms files. The centroids of the most abundant clusters were selected as representative frames and analyzed in terms of interactions established between the peptides and the antibiotic molecules. The surfaces areas of the structures observed were also generated and analyzed, using Vega ZZ version 3.2.0 [32]. Molecular lipophilicity potential (MLP), with a probe radius of 1.5 and mesh size of 0.60, Broto log P, lipole (a measure of the lipophilicity distribution) and virtual log P were also obtained (Table S3) [33].

#### 3.4.2. Docking Studies of Peptides with RND Efflux Pump 4DX5

Inactive peptides **3**, **23** and active peptides **6**, **27** were initially prepared by building their structures using the Fragment Builder tool integrated in Maestro version 11.0.014 (Schrödinger LLC, New York, NY, USA) and the correct ionization states of ionizable groups were set for pH 7 using the Protein Preparation Wizard. A cubic periodic aqueous system (SPC model) with sides 15 Å larger than a molecular system was created for each peptide using the system builder tool, with 0.15 M NaCl concentration, including Na^+^ and Cl^−^ as counter ions. The default Desmond minimization procedure was applied, and simulated annealing was then performed using the OPLS2005 all atoms force field implemented in Desmond and Maestro version 11.0.014 as graphical user interface (Schrödinger, New York, NY, USA). The system was heated at 10 K for 30 ps, 100 K for 100 ps, 300 K for 200 ps, 1000 K for 300 ps, 1000 K for 500 ps and cooled at 300 K for 1000 ps. Short range coulombic interactions were cut off at 9.0 Å. Simulation temperature was regulated with the Berendsen thermostats, with 1.0 ps relaxation time within the NVT ensemble [26]. The equations were integrated with the RESPA integrator, with an inner and outer time steps of 1.0 and 3.0 fs respectively [27,28]. The results were saved as trajectory by storing coordinates and the energies to disk at every 5 ps.

The last frame of the trajectory was then extracted, a 10 Å cubic periodic aqueous system (SPC model) larger in all directions from the molecules was created with the addition of 0.15 M NaCl. Default Desmond minimization was applied as usual and 10 ns MD simulations were performed on the systems using the same force field and software. The cut off value of 9.0 Å was set in calculations of van der Waals and short-range coulombic interactions. Temperature was kept constant at 300 K with the Nose-Hoover thermostats and pressure was maintained with the Martyna–Tobias–Klein barostats within the NPT ensemble [28,29]. The equation of motion was solved with the RESPA integrator, with an inner time step of 2.0 fs and an outer time step of 6.0 fs [27]. The final conformation was extracted and saved as a .mol2 file for docking.

Novobiocin, norfloxacin and PAβN were built using the 3D Sketcher tool implemented in Maestro, and submitted to 10 ns MD simulations in a solvent periodic box 10 Å larger than the size of the molecule in all directions, applying the same parameters described above. The final conformations were extracted from all MD simulations as .mol2 files and used for the docking experiments.

The RND efflux pump (PDB ID 4DX5) was used as a target in the docking simulations. A truncated model containing just the periplasmic domain was prepared for docking with Protein Preparation Wizard implemented in Maestro version 11.0.014 (Schrödinger, New York, NY, USA). This was achieved by deleting all residues except 33-335, 365-871, which contain the distal and proximal binding pockets and the loop regulating the passage of substrates between the two as previously described in literature, as illustrated by Vargiu et al. [20].

The protein model was then subjected to quick prep routine in MOE version 2016.0802 using the default parameters. The binding site was obtained using the site finder function of the software and selecting the largest site proposed. All residues forming the proximal and distal binding pocket as well as the inner loop were included in the proposed binding site, without a need to manually add significant residues.

The set of molecules prepared for docking was imported and submitted to the quick prep routine as well and finally combined into a molecular database (.mdb file).

The six small molecules prepared as .mol2 files were then all docked into the prepared binding site using an induced fit protocol, where the backbone of the receptor is held rigid, while the side chains and the ligand are free to change the conformation. It was chosen to generate a maximum of 15 poses for each ligand, or less if the conformation generated reached a default cut off value of 3.0 Å for the RMSD. Finally, the docked poses obtained were analyzed in terms of docking scores and protein-ligand interaction fingerprint (PLIF).

## Figures and Tables

**Table 1:** Antimicrobial activity of novobiocin in the presence of potentiating peptides at fixed concentrations against susceptible *E. coli* 10,418 and multi-drug resistant clinical isolate *E. coli* G69. A potentiation assay for novobiocin was also performed in the presence of 32 μg/mL of RND efflux pump inhibitor PAβN. All tests were performed in duplicate.

Compound	Sequence-NH_2_	MIC of Novobiocin (μg/mL)			
		*E. coli* NCTC 10418		*E. coli* G69	
**Novobiocin**		32		>128	
	**Added Peptide**	+128 μg/mL	+32 μg/mL	+128 μg/mL	+32 μg/mL
**1**	FRW	NP	NT	NP	NT
**2**	FWR	NP	NT	NP	NT
**3**	WRW	8	64	NP	NT
**4**	WRWR	2	32	NP	NT
**5**	RWRW	2	64	16	64
**6**	WRWRW	0.125	8	16	8
**7**	FRF	NP	NT	NP	NT
**8**	FRFR	NP	NT	8	NP
**9**	RFRF	2	NP	8	128
**10**	FRFRF	2	NT	4	8
**11**	RRFRF	64	NT	64	NT
**12**	WKW	16	64	NP	NT
**13**	WKWK	NP	NT	NP	NT
**14**	KWKW	NP	NT	NP	NT
**15**	WKWKW	4	8	8	128
**16**	FKF	NP	NT	NP	NT
**17**	FKFK	NP	NT	NP	NT
**18**	KFKF	NP	NT	NP	NT
**19**	FKFKF	4	128	8	32
**20**	WRRQRW	4	16	32	NP
**21**	FRRQRF	NP ^†^	NT	NP	NT
**22**	RPRPRPL	NP	NT	NP	NT
**23**	RPWPPR	4	NT	NP	NT
**24**	WKPLPPR	NP	NT	NP	NT
**25**	FKPLPPH	8	NT	NP	NT
**26**	RPPWRPPW	8	64	NP	NT
**27**	RPRRPRLPW	0.125	8	16	16
+64 μg/mL PAβN ^‡^		8		2	
+32 μg/mL PAβN		16		8	

^†^ Non potentiating (NP) indicated no reduction in the MIC of novobiocin was observed; the symbol NT indicates tests were therefore not repeated at a lower concentration of 32 μg/mL. ^‡^ The MIC of novobiocin was also measured in combination with fixed concentrations of 64 μg/mL and 32 μg/mL of the known efflux pump inhibitor PaβN.

**Table 2:** Molecular lipophilicity maps obtained for the most representative complexes and for the free antibiotics.

Complex	Surface Area (Å^2^)	Broto log *P*	Broto Lipole	Virtual log *P*
Novobiocin	968.9 (ds 17.6 Å)	2.3840	1.4807	3.5449
Norfloxacin	556.1 (ds 13.3 Å)	−5.8350	0.4173	−3.0671
WRWRW-NH_2_	1280.5 (ds 20.2 Å)	−12.7310	6.0423	−6.2840
RPRRPRLPW-NH_2_	1861.8 (ds 24.3 Å)	−24.6980	5.0993	−12.2239
Novobiocin—WRWRW-NH_2_ A	2353.1 (ds 27.4 Å)	−5.2310	2.6575	−1.4918
Novobiocin—WRWRW-NH_2_ B	2219.3 (ds 26.6 Å)	−22.3820	1.8365	−9.9088
Norfloxacin—WRWRW-NH_2_	1648.9 (ds 22.9 Å)	−29.8880	1.4519	−14.9976
Novobiocin—RPRRPRLPW-NH_2_	6088.6 (ds 44.0 Å)	−59.7900	1.8135	−24.2018

**Table 3:** Docking scores obtained for the known efflux pump inhibitor PAβN, reference antibiotics novobiocin and norfloxacin, potentiating peptides **6** and **27** and non-potentiating peptide **3**.

Compound	Docking Score
PAβN	−7.3054
Norfloxacin	−5.8704
Novobiocin	−8.7469
RPRRPRLPW (27)	−14.6003
RPWPPR (23)	−11.3160
WRWRW (6)	−12.2049
WRW (3)	−8.1518

**Table 4:** Molar percentage yields.

Compound	Sequence-NH_2_	Molar % Yield	Compound	Sequence-NH_2_	Molar % Yield
**1**	FRW	38	**15**	WKWKW	44
**2**	FWR	42	**16**	FKF	53
**3**	WRW	45	**17**	FKFK	56
**4**	WRWR	49	**18**	KFKF	55
**5**	RWRW	52	**19**	FKFKF	62
**6**	WRWRW	51	**20**	WRRQRW	*
**7**	FRF	55	**21**	FRRQRF	*
**8**	FRFR	58	**22**	RPRPRPL	52
**9**	RFRF	56	**23**	RPWPPR	56
**10**	FRFRF	60	**24**	WKPLPPR	48
**11**	RRFRF	48	**25**	FKPLPPH	*
**12**	WKW	36	**26**	RPPWRPPW	39
**13**	WKWK	39	**27**	RPRRPRLPW	45
**14**	KWKW	41			

* Peptides 20, 21 and 25 were commercial samples from ThermoFisher UK.

## Supplementary Materials

Supplementary Materials can be found at https://www.mdpi.com/1422-0067/21/23/9134/s1.

## Abbreviations

AMP	Antimicrobial peptide
CFU	Colony-forming unit
DMSO	Dimethyl sulfoxide
Fmoc	Fluorenylmethyloxycarbonyl
HBTU	2-(1H-Benzotriazole-1-yl)-1,1,3,3-tetramethyluronium hexafluorophosphate
MLP	Molecular lipophilicity potential
PAβN	Phenylalanine-arginine-β-naphthylamide
PDB	Protein Data Bank
RMSD	Root mean square deviation
RND	Resistance nodulation division
RP-HPLC	Reverse phase high performance liquid chromatography
SPC	Statistical process control
