# Supplementary material for: Synthesis and in Silico Modelling of the Potential Dual Mechanistic Activity of Small Cationic Peptides Potentiating the Antibiotic Novobiocin against Susceptible and Multi-Drug Resistant Escherichia coli
Source: Int J Mol Sci. 2020 Nov 30;21(23):9134. doi: 10.3390/ijms21239134 (PMC7730182; doi:10.3390/ijms21239134)
Supplement: Supplementary file 1 [file ijms-21-09134-s001.pdf]

Supplementary materials for:

Synthesis and *in silico* modelling of the potential dual mechanistic activity of small cationic peptides potentiating the antibiotic novobiocin against susceptible and multi-drug resistant *Escherichia coli*

Ilaria Passarini[1], Pedro Ernesto de Resende[2], Sarah Soares[2], Tadeh Tahmasi[1], Paul Stapleton[2], John Malkinson[2], Mire Zloh[1,3]* and Sharon Rossiter[1]*

[1] School of Life and Medical Sciences, University of Hertfordshire, College Lane, Hatfield, AL10 9AB, UK; i.passarini@herts.ac.uk (I.P.), tadehtahmasi@gmail.com (T.T.), zloh@live.co.uk (M.Z.), s.rossiter@herts.ac.uk (S.R.).

[2] UCL School of Pharmacy, 29-39 Brunswick Square, London WC1N 1AX, UK; pedroderesende@gmail.com (P.R.), sarahapso@gmail.com (S.S.), p.stapleton@ucl.ac.uk (P.S.), j.malkinson@ucl.ac.uk (J.M.).

[3] NanoPuzzle Medicines Design, Stevenage, UK; zloh@live.co.uk

* Correspondence: s.rossiter@herts.ac.uk (S.R.), zloh@live.co.uk (M.Z.)

Contents

1. HPLC data for purified peptides 2

2. Mass spectrometry 10

3. NMR data 12

4. Biological assays 14

5. MD simulation and docking studies. 16

6. Docking against RND efflux pump as a target 18

1. HPLC data for purified peptides

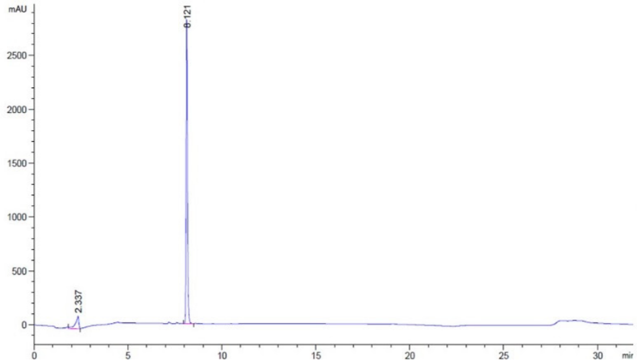

Signal 1: VWD1 A, Wavelength=214 nm

Peak #	RetTime [min]	Type	Width [min]	Area [mAU*s]	Height [mAU]	Area %
1	2.337	VB	0.1581	1443.71143	117.35715	9.5406
2	8.121	BV	0.0740	1.36887e4	2825.92212	90.4594
Totals :				1.51324e4	2943.27927	

Figure S1 Analytical chromatogram of peptide **1** FRW after purification

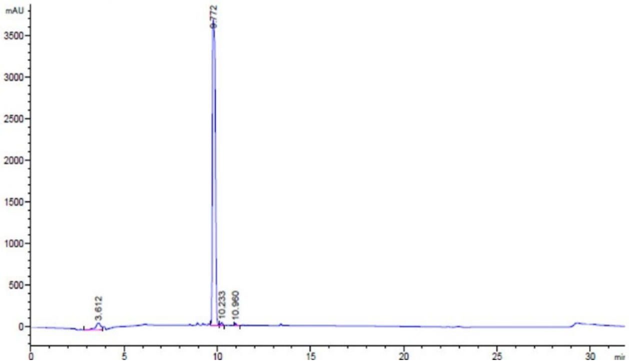

Peak #	RetTime [min]	Type	Width [min]	Area [mAU*s]	Height [mAU]	Area %
1	3.612	BV	0.2907	1633.99963	80.98199	3.8755
2	9.772	VV	0.1818	4.01888e4	3664.53638	95.3194
3	10.233	VV	0.0927	217.31383	33.68820	0.5154
4	10.960	VB	0.0694	122.12791	26.68702	0.2897
Totals :				4.21622e4	3805.89359	

Figure S2. Analytical chromatogram of peptide **2** FWR after purification

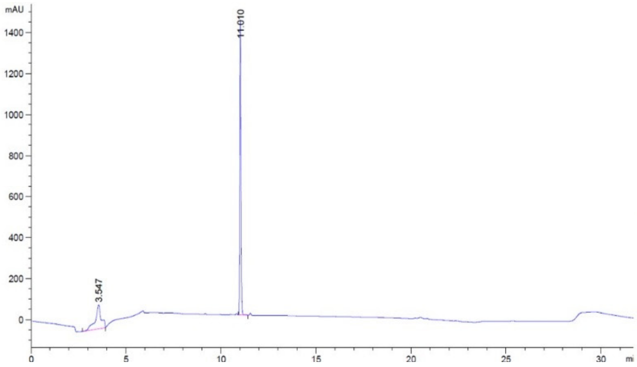

Signal 1: VWD1 A, Wavelength=214 nm

Peak #	RetTime [min]	Type	Width [min]	Area [mAU*s]	Height [mAU]	Area %
1	3.547	BB	0.2962	2482.32422	116.02134	28.4073
2	11.010	BH	0.0896	6256.01416	1334.73511	71.5927
Totals :				8738.33838	1450.75645	

Figure S3. Analytical chromatogram of peptide **3** WRW after purification

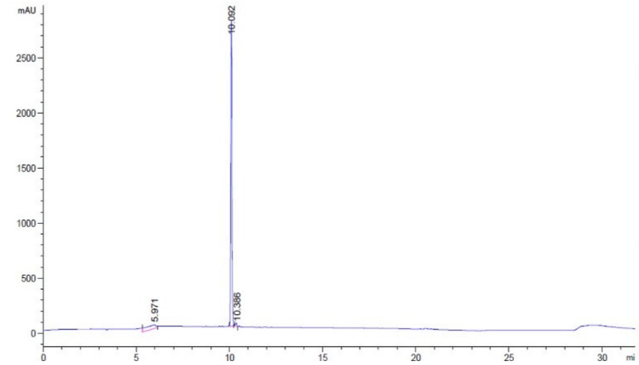

Signal 1: VWD1 A, Wavelength=214 nm

Peak #	RetTime [min]	Type	Width [min]	Area [mAU*s]	Height [mAU]	Area %
1	5.971	MM	0.5287	1507.56763	37.04300	10.5261
2	10.092	MM	0.0763	1.26422e4	2761.18237	88.2704
3	10.386	MM	0.1202	172.36469	23.90258	1.2035
Totals :				1.43222e4	2822.12796	

Figure S4. Analytical chromatogram of peptide **4** WRWR after purification

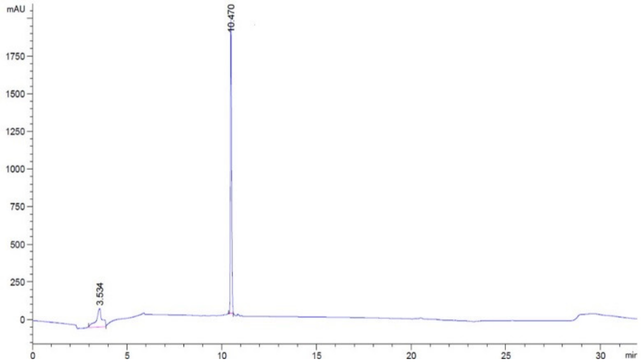

Signal 1: VWD1 A, Wavelength=214 nm

Peak #	RetTime [min]	Type	Width [min]	Area [mAU*s]	Height [mAU]	Area %
1	3.534	MM	0.3518	2604.16943	123.37173	23.2041
2	10.470	MM	0.0745	8618.70898	1928.99841	76.7959
Totals :				1.12229e4	2052.37014	

Figure S5. Analytical chromatogram of peptide **5** RWRW after purification

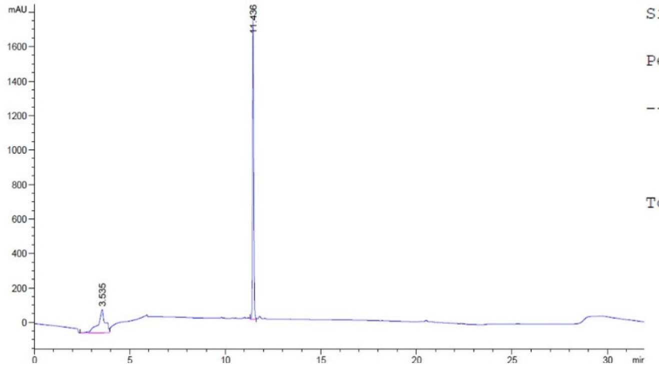

Signal 1: VWD1 A, Wavelength=214 nm

Peak #	RetTime [min]	Type	Width [min]	Area [mAU*s]	Height [mAU]	Area %
1	3.535	BV	0.3480	3475.84937	134.52489	31.6358
2	11.436	MM	0.0696	7511.22656	1797.97180	68.3642
Totals :				1.09871e4	1932.49669	

Figure S6. Analytical chromatogram of peptide **6** WRWRW after purification

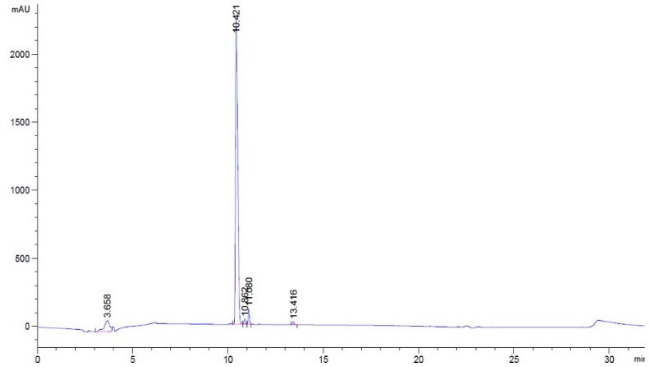

Signal 1: VWD1 A, Wavelength=214 nm

Peak #	RetTime [min]	Type	Width [min]	Area [mAU*s]	Height [mAU]	Area %
1	3.658	BV	0.2942	1634.23010	80.31004	7.6391
2	10.421	VV	0.1395	1.89522e4	2239.71509	88.5901
3	10.862	VB	0.0734	194.77809	40.66743	0.9105
4	11.080	BV	0.0665	487.15958	112.53944	2.2772
5	13.416	VV	0.0813	124.75549	23.33349	0.5832
Totals :				2.13931e4	2496.56549	

Figure S7. Analytical chromatogram of peptide **7** FRF after purification

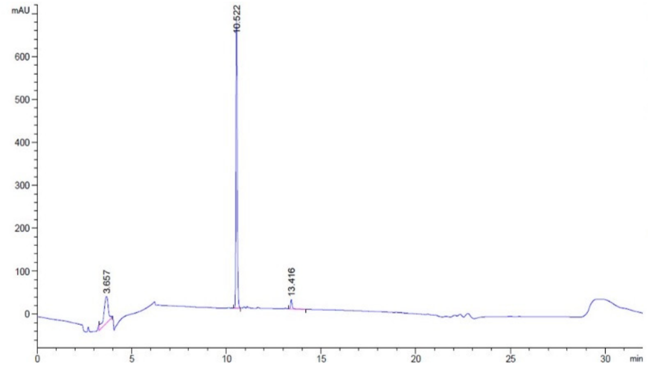

Signal 1: VWD1 A, Wavelength=214 nm

Peak #	RetTime [min]	Type	Width [min]	Area [mAU*s]	Height [mAU]	Area %
1	3.657	MM	0.2798	1038.44873	61.86236	22.7338
2	10.522	BV	0.0797	3403.15576	669.78851	74.5021
3	13.416	VB	0.0862	126.25941	21.88336	2.7641
Totals :				4567.86391	753.53424	

Figure S8. Analytical chromatogram of peptide **8** FRFR after purification

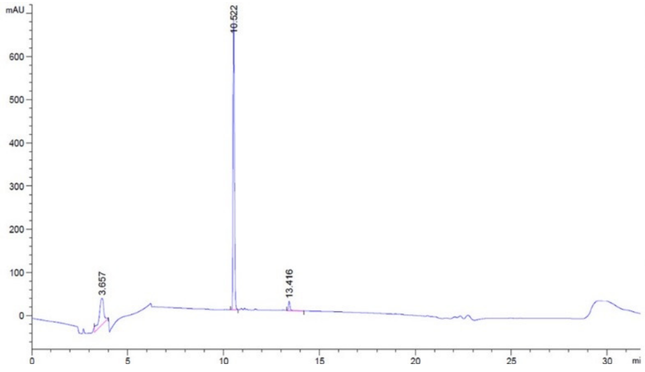

Signal 1: VWD1 A, Wavelength=214 nm

Peak #	RetTime [min]	Type	Width [min]	Area [mAU*s]	Height [mAU]	Area %
1	3.627	MM	0.2392	830.98846	57.90961	15.8610
2	10.032	VB	0.0771	4106.10791	823.49475	78.3727
3	10.503	BV	0.0978	201.95804	28.29336	3.8547
4	13.426	MM	0.0761	100.14973	21.92471	1.9115
Totals :				5239.20415	931.62242	

Figure S9. Analytical chromatogram of peptide **9** RFRF after purification

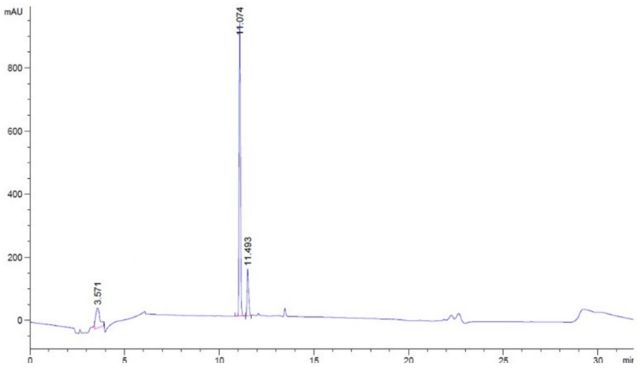

Signal 1: VWD1 A, Wavelength=214 nm

Peak #	RetTime [min]	Type	Width [min]	Area [mAU*s]	Height [mAU]	Area %
1	3.571	MM	0.2621	994.92236	63.26732	15.3726
2	11.074	BB	0.0786	4652.24512	932.90912	71.8822
3	11.493	BB	0.0791	824.87610	149.14400	12.7452
Totals :				6472.04358	1145.32044	

Figure S10. Analytical chromatogram of peptide **10** FRFRF after purification

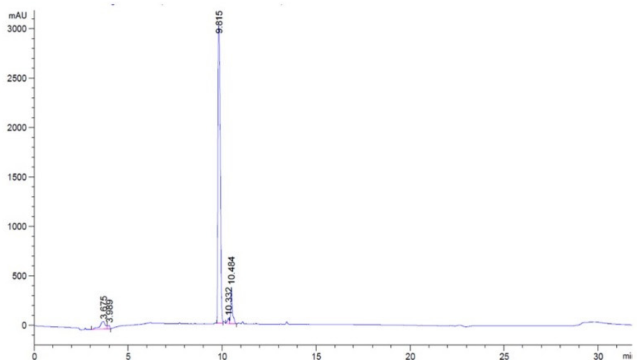

Peak #	RetTime [min]	Type	Width [min]	Area [mAU*s]	Height [mAU]	Area %
1	3.675	BV	0.3036	1550.95813	74.63941	5.1923
2	3.989	VB	0.1136	268.44482	31.15097	0.8987
3	9.815	BV	0.1392	2.54166e4	3012.19482	85.0893
4	10.332	BV	0.0887	343.29535	55.09296	1.1493
5	10.484	VB	0.0918	2291.20508	366.73273	7.6705
Totals :				2.98705e4	3539.81089	

Figure S11. Analytical chromatogram of peptide **11** RRFRF after purification

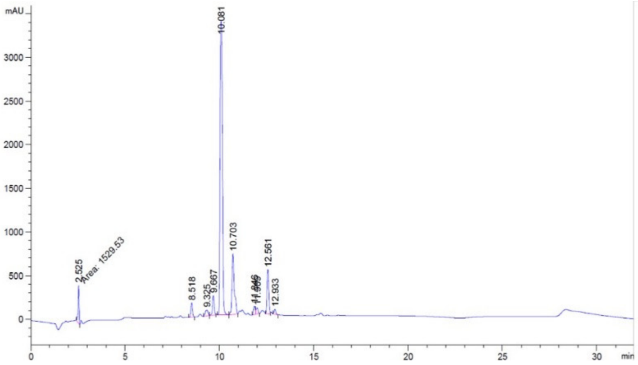

Signal 1: VWD1 A, Wavelength=214 nm

Peak #	RetTime [min]	Type	Width [min]	Area [mAU*s]	Height [mAU]	Area %
1	2.525	MM	0.0582	1529.53027	437.83850	3.4129
2	8.518	BV	0.0961	984.09290	164.36742	2.1958
3	9.325	BB	0.1524	697.80206	66.01129	1.5570
4	9.667	BV	0.0958	1352.32690	222.14011	3.0175
5	10.081	BB	0.1431	2.95355e4	3366.41016	65.9034
6	10.703	BB	0.1281	5954.03320	691.57269	13.2854
7	11.846	BV	0.1072	615.76367	92.03642	1.3740
8	11.989	VB	0.1074	483.05524	69.37994	1.0779
9	12.561	BV	0.1068	3311.66260	506.86240	7.3894
10	12.933	BB	0.1049	352.53879	55.35902	0.7866
Totals :				4.48163e4	5671.97794	

Figure S12. Analytical chromatogram of peptide **12** WKW after purification

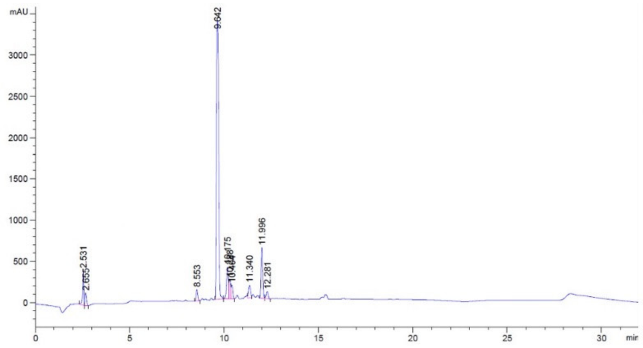

Signal 1: VWD1 A, Wavelength=214 nm

Peak #	RetTime [min]	Type	Width [min]	Area [mAU*s]	Height [mAU]	Area %
1	2.531	MM	0.0593	1500.13196	421.53333	3.6876
2	2.655	MM	0.0905	831.27173	153.14037	2.0434
3	8.553	BB	0.0968	826.84760	136.55688	2.0325
4	9.642	BB	0.1294	2.68516e4	3369.99292	66.0057
5	10.175	BV	0.1066	2680.94946	388.85199	6.5902
6	10.288	VV	0.0876	1355.01965	235.22331	3.3309
7	10.404	VB	0.0922	980.83868	165.97980	2.4111
8	11.340	VV	0.1187	1137.13562	153.11877	2.7953
9	11.996	BV	0.1008	3855.99902	615.25745	9.4787
10	12.281	VB	0.1178	660.92773	86.91357	1.6247
Totals :				4.06807e4	5726.56838	

Figure S13. Analytical chromatogram of peptide **13** WKWK after purification

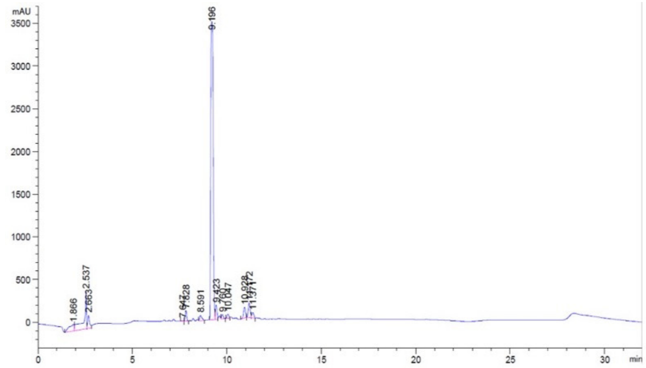

Signal 1: VWD1 A, Wavelength=214 nm

Peak #	RetTime [min]	Type	Width [min]	Area [mAU*s]	Height [mAU]	Area %
1	1.866	BV	0.2547	1476.03223	74.89606	3.4011
2	2.537	VV	0.1175	4067.61865	442.05624	9.3726
3	2.663	VV	0.1064	1084.50598	152.31467	2.4989
4	7.647	VB	0.0900	10.63023	1.99073	0.0245
5	7.828	BV	0.0831	628.50677	119.77664	1.4482
6	8.591	VB	0.1240	514.69165	60.48044	1.1859
7	9.196	BV	0.1486	3.13726e4	3488.34009	72.2887
8	9.423	VV	0.0942	996.16766	167.29364	2.2954
9	9.760	VB	0.1002	315.75885	49.77328	0.7276
10	10.047	BV	0.1198	378.58676	49.50746	0.8723
11	10.928	BB	0.1086	887.00470	127.92730	2.0438
12	11.172	BV	0.1134	1215.34790	171.26324	2.8004
13	11.371	VB	0.1180	451.61481	61.28916	1.0406
Totals :				4.33991e4	4966.90895	

Figure S14. Analytical chromatogram of peptide **14** KWKW after purification

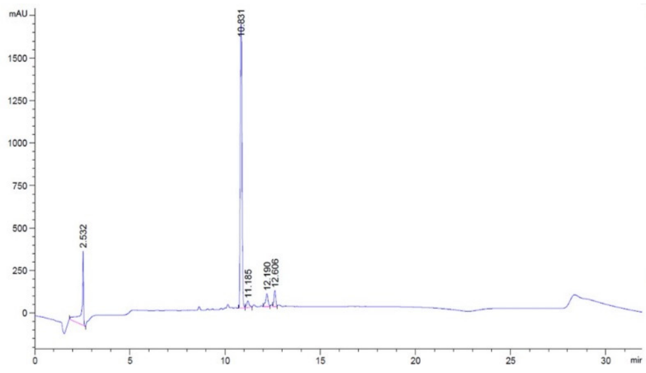

Signal 1: VWD1 A, Wavelength=214 nm

Peak #	RetTime [min]	Type	Width [min]	Area [mAU*s]	Height [mAU]	Area %
1	2.532	MM	0.1061	2792.29395	438.71683	18.5950
2	10.831	BV	0.1027	1.05454e4	1672.92603	70.2259
3	11.185	VV	0.1389	363.25446	38.09521	2.4191
4	12.190	BV	0.1357	692.93384	76.91332	4.6145
5	12.606	VV	0.1055	622.51282	95.13507	4.1456
Totals :				1.50164e4	2321.78646	

Figure S15. Analytical chromatogram of peptide **15** WKWKW after purification

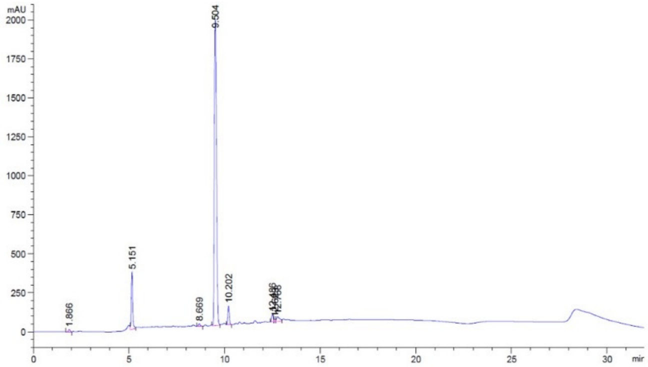

Signal 1: VWD1 A, Wavelength=214 nm

Peak #	RetTime [min]	Type	Width [min]	Area [mAU*s]	Height [mAU]	Area %
1	1.866	BV	0.0839	84.76854	15.93672	0.4536
2	5.151	VV	0.0776	1850.84045	367.98636	9.9042
3	8.669	BB	0.1015	103.88407	15.78389	0.5559
4	9.504	VV	0.1257	1.52438e4	1960.18066	81.5723
5	10.202	BB	0.0896	672.42822	120.97612	3.5983
6	12.486	BV	0.0986	294.87820	47.49854	1.5779
7	12.623	VV	0.0789	84.28813	15.64625	0.4510
8	12.768	VV	0.1667	352.58560	30.53461	1.8868
Totals :				1.86874e4	2574.54315	

Figure S16. Analytical chromatogram of peptide **16** FKF after purification

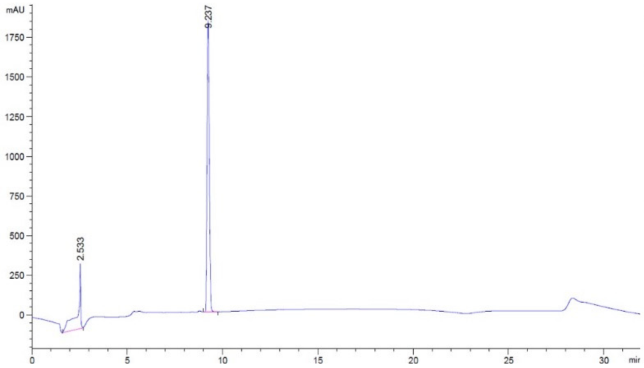

Signal 1: VWD1 A, Wavelength=214 nm

Peak #	RetTime [min]	Type	Width [min]	Area [mAU*s]	Height [mAU]	Area %
1	2.533	MM	0.1968	4835.22266	409.47208	24.4069
2	9.237	BV	0.1315	1.49756e4	1837.84167	75.5931
Totals :				1.98109e4	2247.31375	

Figure S17. Analytical chromatogram of peptide **17** FKFK after purification

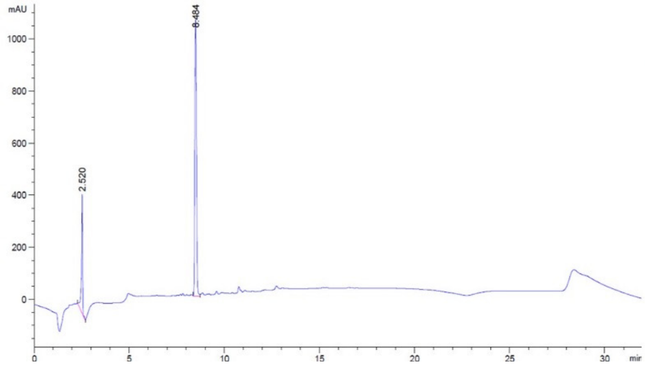

Signal 1: VWD1 A, Wavelength=214 nm

Peak #	RetTime [min]	Type	Width [min]	Area [mAU*s]	Height [mAU]	Area %
1	2.520	MM	0.0642	1762.35559	457.51413	21.7748
2	8.484	MM	0.0993	6331.19629	1063.06738	78.2252
Totals :				8093.55188	1520.58151	

Figure S18. Analytical chromatogram of peptide **18** KFKF after purification

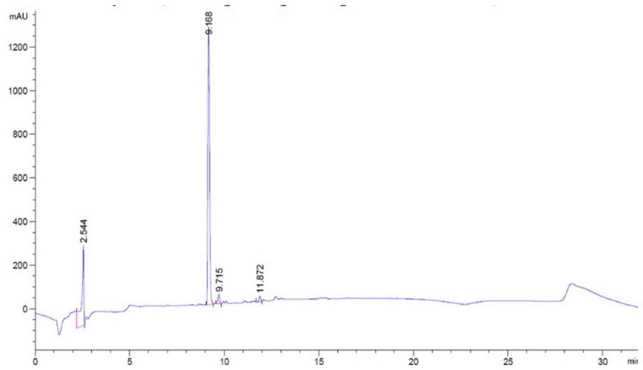

Signal 1: VWD1 A, Wavelength=214 nm

Peak #	RetTime [min]	Type	Width [min]	Area [mAU*s]	Height [mAU]	Area %
1	2.544	VB	0.1006	2800.11548	367.29449	24.6959
2	9.168	VV	0.1001	8071.13428	1274.17676	71.1843
3	9.715	BB	0.1038	309.33200	44.82362	2.7282
4	11.872	BV	0.0971	157.77873	25.96002	1.3915
Totals :				1.13384e4	1712.25489	

Figure S19. Analytical chromatogram of peptide **19** FKFKF after purification

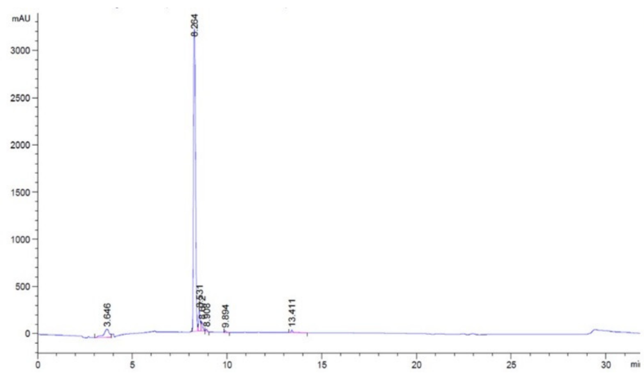

Signal 1: VWD1 A, Wavelength=214 nm

Peak #	RetTime [min]	Type	Width [min]	Area [mAU*s]	Height [mAU]	Area %
1	3.646	BV	0.2906	1710.91528	84.82717	6.1205
2	8.264	BV	0.1207	2.39637e4	3204.34912	85.7259
3	8.531	VV	0.0935	1373.94714	219.22281	4.9150
4	8.672	VB	0.0844	544.01465	94.85023	1.9461
5	8.908	BB	0.0765	104.13011	21.08602	0.3725
6	9.894	VV	0.0719	83.90994	17.99425	0.3002
7	13.411	VV	0.0912	173.24252	27.95472	0.6197
Totals :				2.79539e4	3670.28433	

Figure S20. Analytical chromatogram of peptide **22** RPRPRPL after purification

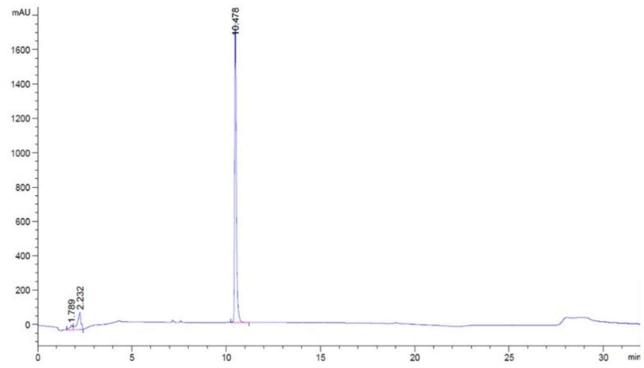

Signal 1: VWD1 A, Wavelength=214 nm

Peak #	RetTime [min]	Type	Width [min]	Area [mAU*s]	Height [mAU]	Area %
1	1.789	VV	0.1463	306.02731	27.41236	2.5113
2	2.232	VB	0.1640	1253.87427	99.82978	10.2895
3	10.478	VB	0.0928	1.06260e4	1746.31323	87.1992
Totals :				1.21859e4	1873.55537	

Figure S21. Analytical chromatogram of peptide **23** RPWPPR after purification

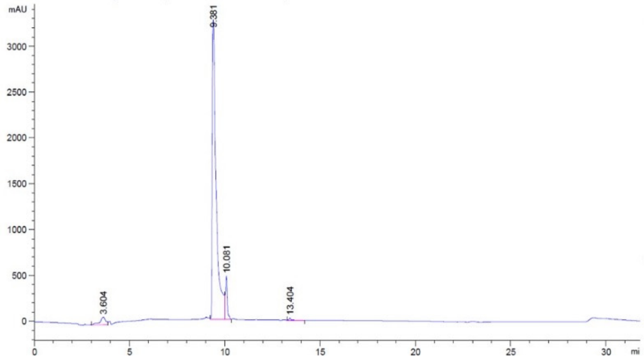

Signal 1: VWD1 A, Wavelength=214 nm

Peak #	RetTime [min]	Type	Width [min]	Area [mAU*s]	Height [mAU]	Area %
1	3.604	VV	0.3029	1724.44727	81.26237	3.3311
2	9.381	VV	0.2034	4.64130e4	3271.01392	89.6549
3	10.081	VB	0.1019	3476.14819	472.36581	6.7148
4	13.404	VB	0.0874	154.88770	26.37369	0.2992
Totals :				5.17685e4	3851.01579	

Figure S22. Analytical chromatogram of peptide **24** WKPLPPR after purification

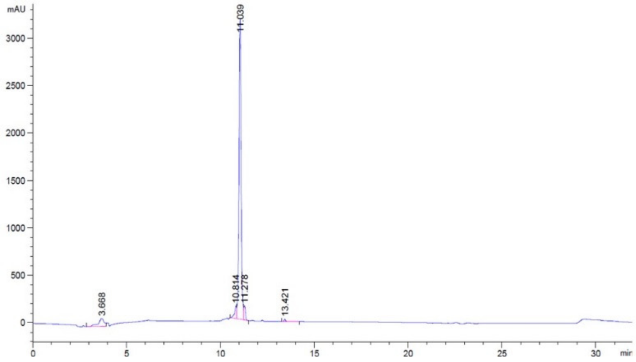

Signal 1: VWD1 A, Wavelength=214 nm

Peak #	RetTime [min]	Type	Width [min]	Area [mAU*s]	Height [mAU]	Area %
1	3.668	VV	0.3061	1749.40491	81.37838	6.0382
2	10.814	BV	0.0951	928.01221	134.45966	3.2031
3	11.039	VV	0.1225	2.53099e4	3163.26563	87.3590
4	11.278	VB	0.0838	836.74420	150.46481	2.8881
5	13.421	VV	0.0880	148.23792	25.03801	0.5117
Totals :				2.89723e4	3554.60648	

Figure S23. Analytical chromatogram of peptide **26** RPPWRPPW after purification

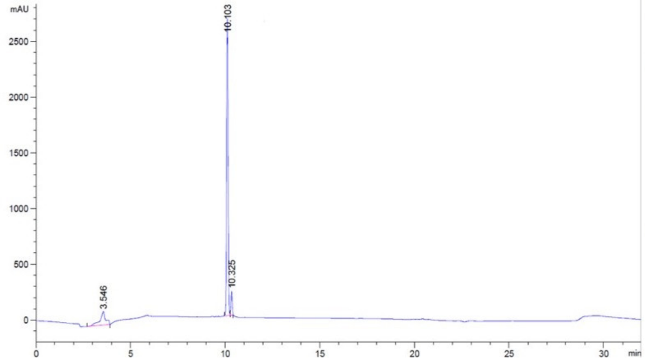

Signal 1: VWD1 A, Wavelength=214 nm

Peak #	RetTime [min]	Type	Width [min]	Area [mAU*s]	Height [mAU]	Area %
1	3.546	BB	0.2928	2539.88403	120.35387	14.0996
2	10.103	MM	0.0920	1.43976e4	2607.43164	79.9250
3	10.325	MM	0.0832	1076.40088	215.57240	5.9754
Totals :				1.80139e4	2943.35791	

Figure S24. Analytical chromatogram of peptide **27** RPRRPRLPW after purification

2. Mass spectrometry

Synthesised peptides were further characterised through mass spectrometry (LC-MS) (Table S2). Total ion chromatograms were obtained on a Varian Prostar triple-quad LC-MS with integrated ESI detector. All pure samples were initially dissolved in 0.02% v/v trifluoroacetic acid in water and were then eluted through a C18-2 5 μm, 250x4.6 mm Temesil™ HPLC column. A mixture of 0.1% v/v formic acid in water (solvent A) and 0.1% v/v formic acid in acetonitrile (solvent B) was used as mobile phase with the following gradient: 10% to 90% B over 20 minutes, maintain 90% B for 5 minutes and then back at 10% B over 7 minutes, with flow rate set at 1 mL/min. The mass of the main peak corresponding to the molecular ion was confirmed with a Varian triple-quad mass spectrometer integrated with ESI detector with voltage capillary set at 80.000 V.

Table S1. *m/z* ratios and LC-MS retention times of synthesised peptides

	Sequence	Molecular mass / amu	Basic residues	Elution time / min	Observed *m/z* (100% abundance unless stated)
1	FRW	506.3	1	6.60	507.4 $[M + H]^{+}$
2	FWR	506.3	1	5.48	507.3 $[M + H]^{+}$
3	WRW	545.3	1	6.91	546.3 $[M + H]^{+}$
4	WRWR	701.4	2	5.42	351.8, $[M + 2H]^{2+}$, 702.4, 7%, $[M + H]^{+}$
5	RWRW	701.4	2	5.85	351.9 $[M + 2H]^{2+}$
6	WRWRW	887.5	2	7.16	444.9 $[M + 2H]^{2+}$
7	FRF	467.3	1	6.18	468.4 $[M + H]^{+}$
8	FRFR	623.4	2	4.75	312.8 $[M + 2H]^{2+}$
9	RFRF	623.4	2	5.11	312.8 $[M + 2H]^{2+}$, 624.4, 40% $[M + H]^{+}$
10	FRFRF	770.4	2	7.11	386.5 $[M + 2H]^{2+}$
11	RRFRF	779.5	3	4.75	261.4, $[M + 3H]^{3+}$, 391.4, $[M + 2H]^{2+}$
12	WKW	517.3	1	6.75	518.30 $[M + H]^{+}$
13	WKWK	645.4	2	5.35	323.5 $[M + 2H]^{2+}$, 646.5, 20% $[M + H]^{+}$
14	KWKW	645.4	2	5.83	324.0 $[M + 2H]^{2+}$
15	WKWKW	831.5	2	6.98	417.0 $[M + 2H]^{2+}$
16	FKF	439.3	1	6.14	440.2 $[M + H]^{+}$
17	FKFK	567.4	2	4.48	285.0 $[M + 2H]^{2+}$, 568.5, 15% $[M + H]^{+}$
18	KFKF	567.4	2	5.19	284.9 $[M + 2H]^{2+}$, 568.5, 35% $[M + H]^{+}$
19	FKFKF	714.4	2	6.56	358.5 $[M + 2H]^{2+}$, 715.7, 10% $[M + H]^{+}$
22	RPRPRPL	889.6	3	3.06	297.8 $[M + 3H]^{3+}$
23	RPWPPR	806.5	2	4.83	404.5 $[M + 2H]^{2+}$
24	WKPLPPR	891.5	2	5.10	447.30 $[M + 2H]^{2+}$, 892.7, 10% $[M + H]^{+}$
26	RPPWRPPW	1089.6	2	7.13	546.0 $[M + 2H]^{2+}$
27	RPRRPRLPW	1231.8	4	4.93	411.7 $[M + 3H]^{3+}$, 617.0, 35% $[M + 2H]^{2+}$

3. NMR data

Peptides **6** and **27** were selected for further investigation and were analysed using NMR spectroscopy, to confirm the expected sequence and connectivity of the residues (Figures S27 and 28; Tables S6 and S7).

^{1}H, NOESY and TOCSY NMR were acquired in a 10% v/v D_2O solution in water on a Bruker Avance 500 MHz spectrometer.

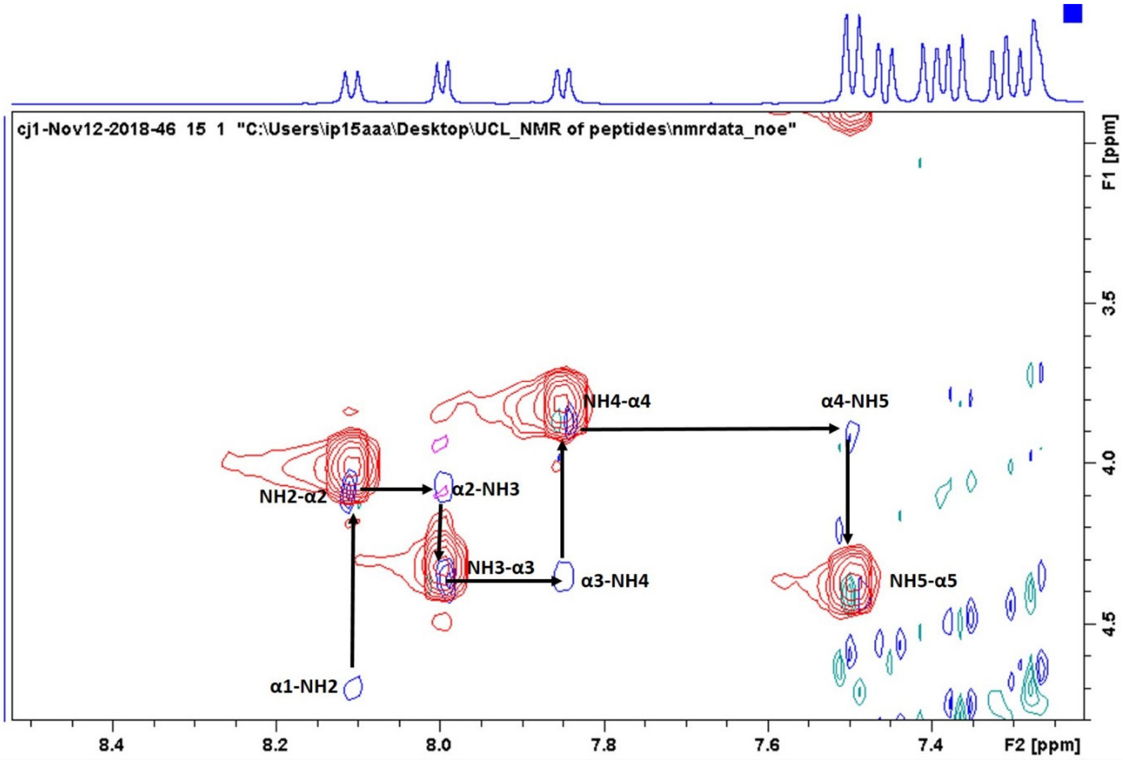

Figure S25. Overlay of the aromatic regions obtained from 2D ^{1}H-^{1}H TOCSY (red) and NOESY (blue) NMR spectra of peptide **6** (WRWRW), with relevant connectivity indicated by black arrows.

Table S2. Assignments of ^{1}H NMR spectra of peptide 6 (WRWRW) based on the overlapping of 2D ^{1}H-^{1}H TOCSY and NOESY spectra.

**Residue**	**H-Nα**	**H-α**	**H-β**	**H-γ**	**H-δ**	**Other**
**Trp1**		**4.72**				**6.95-7.45 (Ar) 10.9 (N$^\alpha$H)**
**Arg2**	**8.10**	**4.01**	**1.47; 1.40**	**1.24**	**2.55**	
**Trp3**	**7.99**	**4.33**	**2.61**			**6.95-7.45 (Ar) 10.9 (N$^\alpha$H)**
**Arg4**	**7.84**	**3.80**	**1.36; 1.30**	**1.05**	**2.43**	
**Trp5**	**7.51**	**4.39**	**2.86; 2.63**			**6.95-7.45 (Ar) 10.9 (N$^\alpha$H)**

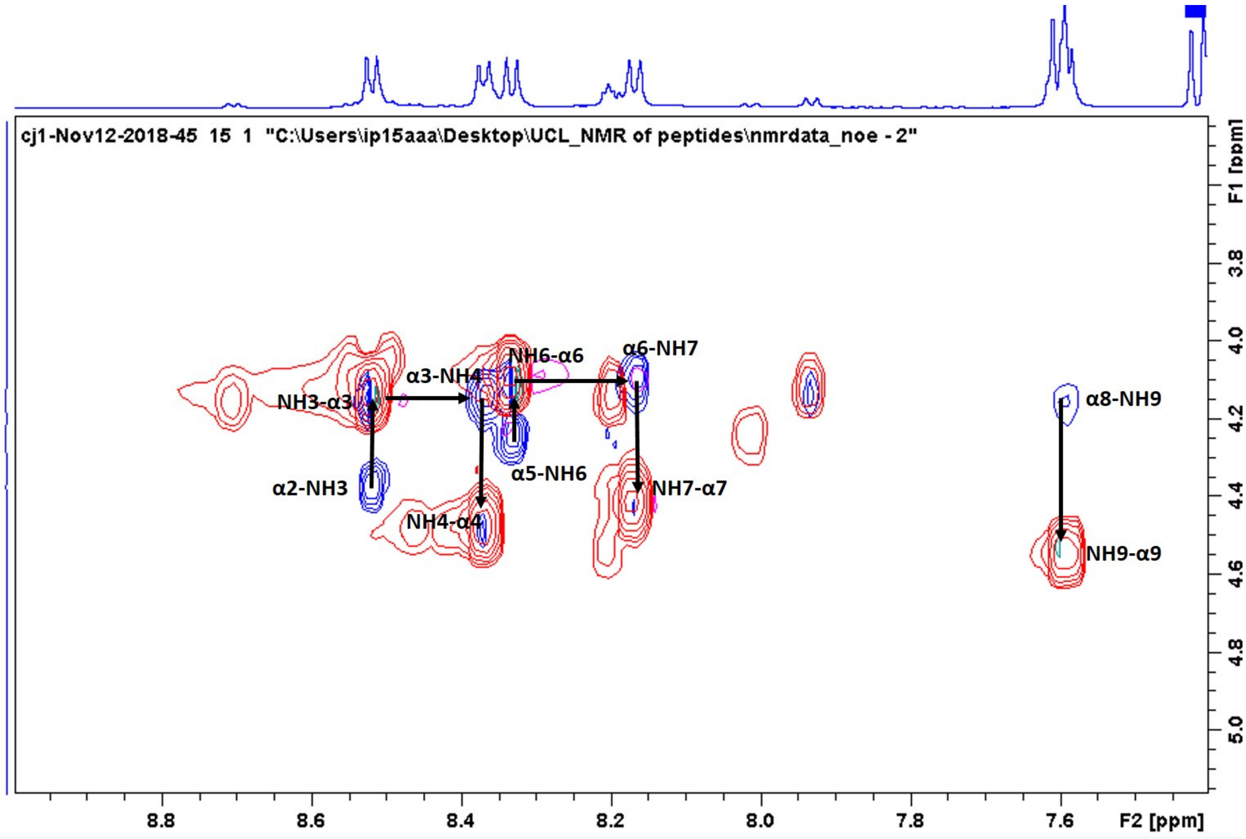

Figure S26. Overlay of the aromatic regions obtained from 2D ^{1}H-^{1}H TOCSY (red) and NOESY (blue) NMR spectra of sample **27** (RPRRPRLPW), with the relevant connectivity indicated by black arrows.

Table S3. Assignment of 1H NMR spectra of peptide **27** (RPRRPRLPW) based on the overlapping of TOCSY and NOESY experiments.

Residue	H-Nα	H-α	H-β	H-γ	H-δ	Other
**Arg 1**	**Not visible**					
**Pro 2**		**4.37**	**2.25; 1.92**	**2.22; 1.77**	**3.66; 3.47**	
**Arg 3**	**8.52**	**4.14**	**1.67**	**1.54**	**2.88**	
**Arg 4**	**8.37**	**4.48**	**1.92**	**1.75**	**3.03**	
**Pro 5**		**4.24**				
**Arg 6**	**8.33**	**4.11**	**1.84**	**1.70**	**2.93**	
**Leu 7**	**8.17**	**4.42**	**1.47; 1.37**	**1.16**	**0.80; 0.76**	
**Pro 8**		**4.15**				
**Trp 9**	**7.59**	**4.52**	**2.96**			**6.92-7.40 Ar) 10.9 (N$^{\alpha}$H)**

4. Biological assays

Table S4. Minimum inhibitory concentration (MIC) of synthesised peptides and of reference antibiotics novobiocin and norfloxacin.

**MIC (µg/mL)**			
		E. coli [illegible]	*S. aureus* [illegible]
**Compound**	**Sequence**		
**Novobiocin**		**32**	**4**
**Norfloxacin**		**0.062**	**2**
**1**	**FRW**	**>128**	**>128**
**2**	**FWR**	**>128**	**>128**
**3**	**WRW**	**>128**	**>128**
**4**	**WRWR**	**>128**	**>128**
**5**	**RWRW**	**>128**	**>128**
**6**	**WRWRW**	**64**	**64**
**7**	**FRF**	**>128**	**>128**
**8**	**FRFR**	**>128**	**>128**
**9**	**RFRF**	**>128**	**>128**
**10**	**FRFRF**	**>128**	**>128**
**11**	**RRFRF**	**>128**	**>128**
**12**	**WKW**	**>128**	**>128**
**13**	**WKWK**	**>128**	**>128**
**14**	**KWKW**	**>128**	**>128**
**15**	**WKWKW**	**>128**	**>128**
**16**	**FKF**	**>128**	**>128**
**17**	**FKFK**	**>128**	**>128**
**18**	**KFKF**	**>128**	**>128**
**19**	**FKFKF**	**>128**	**>128**
**20**	**WRRQRW**	**>128**	**>128**
**21**	**FRRQRF**	**>128**	**>128**
**22**	**RPRPRPL**	**>128**	**>128**
**23**	**RPWPPR**	**>128**	**>128**
**24**	**WKPLPPR**	**>128**	**>128**
**25**	**FKPLPPH**	**>128**	**>128**
**26**	**RPPWRPPW**	**>128**	**>128**
**27**	**RPRRPRLPW**	**128**	**>128**

Table S5. Antimicrobial activity of norfloxacin in presence of peptides which showed some activity in potentiating novobiocin. Assays were performed at fixed concentration of 128 μg/mL against susceptible *E. coli* 10418 and multidrug resistant clinical isolate *E. coli* G69. Potentiation assay for norfloxacin was also performed in presence of 64 μg/mL of RND efflux pump inhibitor PAβN. All tests were repeated in duplicate.

**Compound**	**Sequence**	**MIC of norfloxacin (μg/mL)**	
		***E. coli* 10418**	***E. coli* G69**
**Norfloxacin**		**0.062**	**>128**
		**+128 μg/mL of peptide**	
1	FRW	0.062	>128
2	FWR	0.062	>128
3	WRW	0.062	>128
4	WRWR	0.062	>128
5	RWRW	0.062	>128
6	WRWRW	0.062	>128
7	FRF	0.062	>128
9	RFRF	0.062	>128
12	WKW	0.062	>128
15	WKWKW	0.125	>128
16	FKF	0.062	>128
20	WRRQRW	0.062	>128
21	FRRQRF	0.062	>128
22	RPRPRPL	0.062	>128
23	RPWPPR	0.062	>128
24	WKPLPPR	0.062	>128
25	FKPLPPH	0.062	>128
26	RPPWRPPW	0.62	>128
27	RPRRPRLPW	0.062	>128
**PAβN‡**		0.125	>128

‡ The MIC of norfloxacin was also measured in combination with 64 μg/mL of the known efflux pump inhibitor PaβN but no potentiation was observed, so the assay was not repeated at lower concentrations.

5. MD simulation and docking studies.

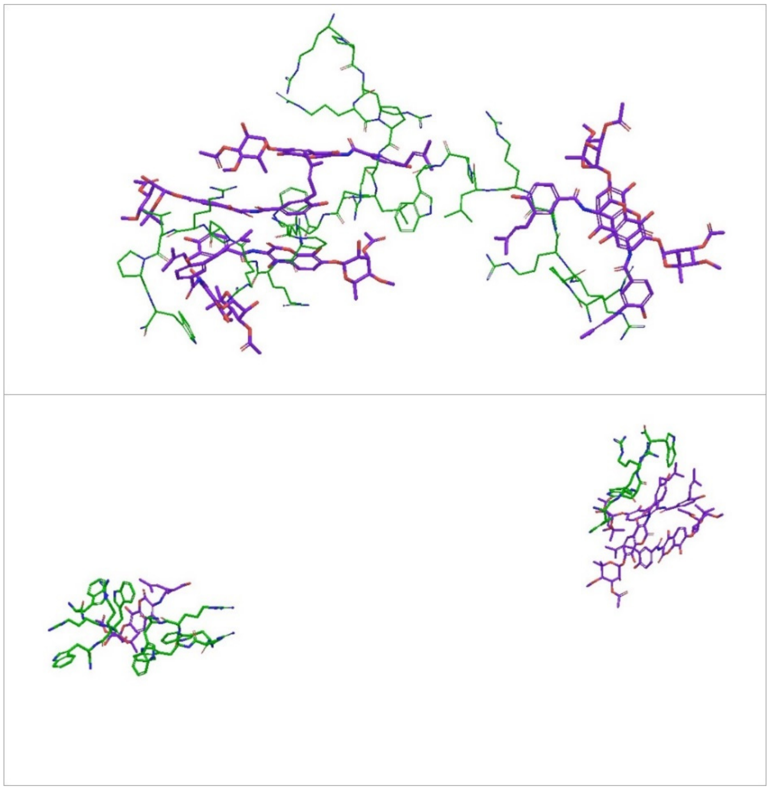

Figure S27. Top: the representative frame of the most populated cluster obtained from the trajectory of 50 ns MD simulation of a system containing peptide **27** (RPRRPRLPW-NH_2, green) and antibiotic novobiocin (purple). All molecules are involved in the formation of one major complex; Bottom: the representative frame of the most populated cluster obtained from the trajectory of 50 ns MD simulation of a system containing peptide 6 (WRWRW-NH_2, green) and novobiocin (purple). All molecules are involved in the formation of two complexes.

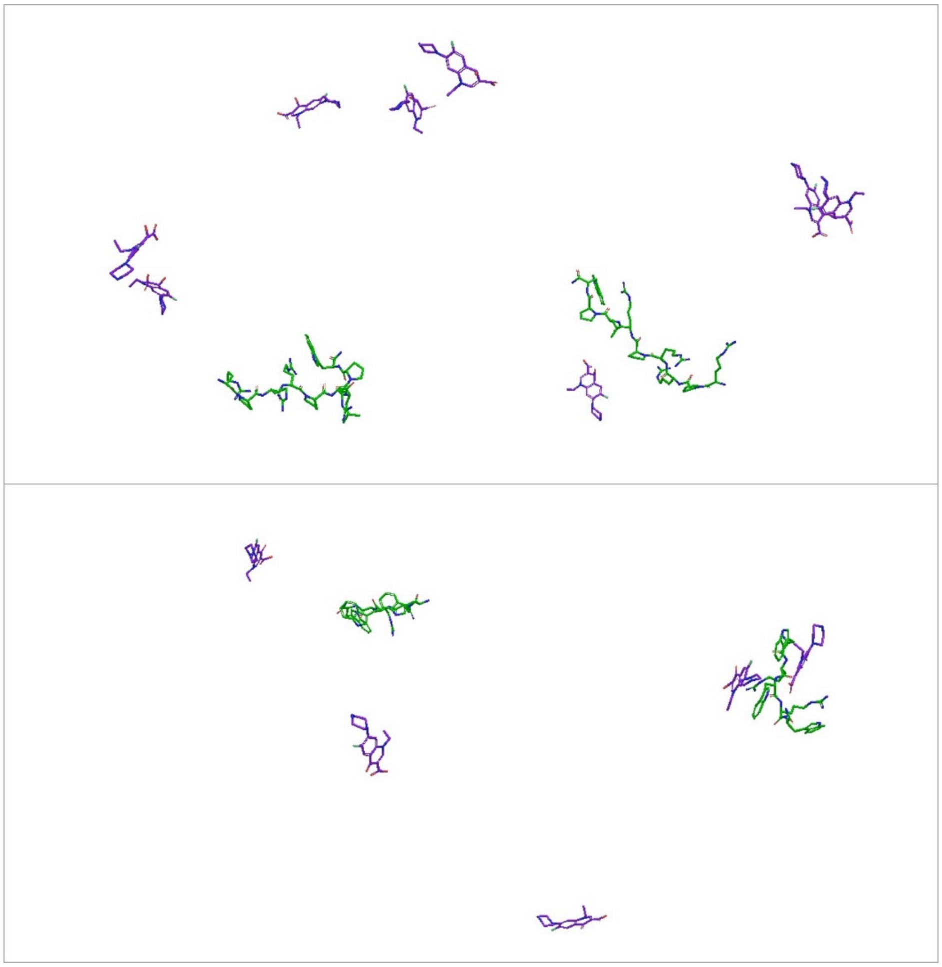

Figure S28. Top: representative frame of the most populated cluster obtained from the trajectory of 50 ns MD simulation of a system containing peptide **27** (RPRRPRLPW-NH_2, green) and antibiotic norfloxacin (purple). No complexes are formed. Bottom: representative frame of the most populated cluster obtained from the trajectory of 50 ns MD simulation of a system containing peptide **6** (WRWRW-NH_2, green) and norfloxacin (purple). Whilst the formation of a complex among two molecules of antibiotic and one of peptide can still be seen, the other three norfloxacin molecules are still scattered throughout the system.

6. Docking against RND efflux pump as a target

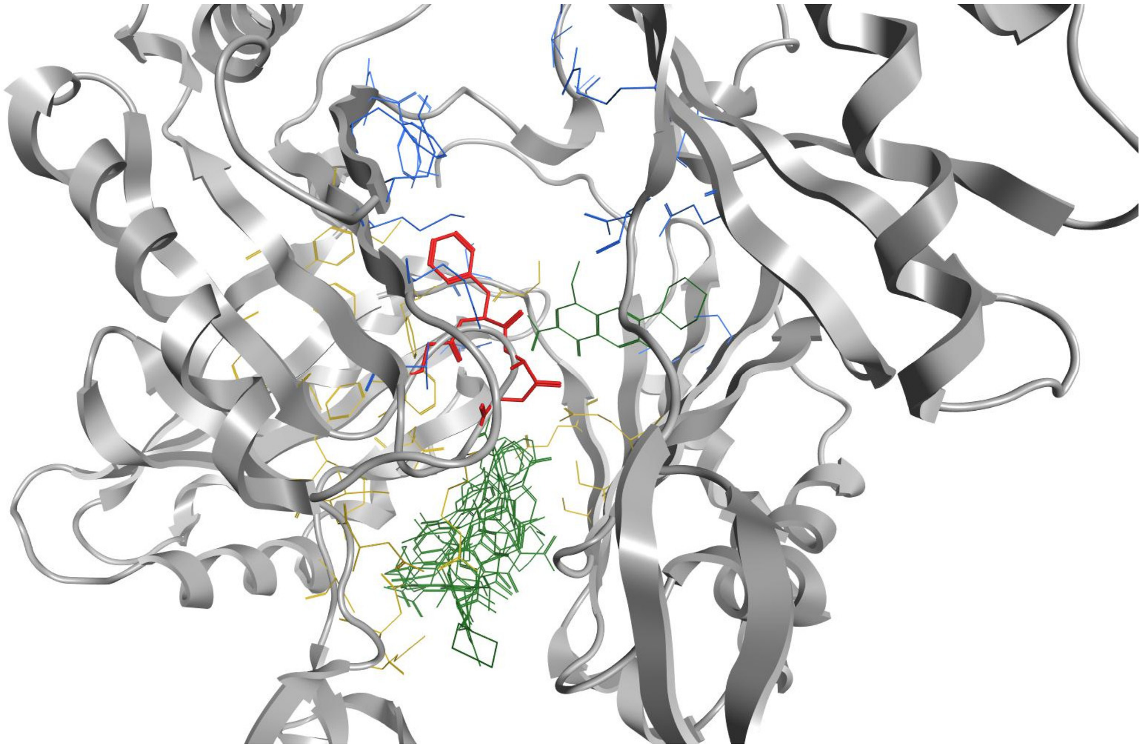

Figure S29. All poses of norfloxacin are found in the distal binding pocket (yellow sticks), with the exception of the most favourable one which is in the proximal binding pocket (blue sticks) instead, also establishing interaction with the glycine loop (red thick sticks).

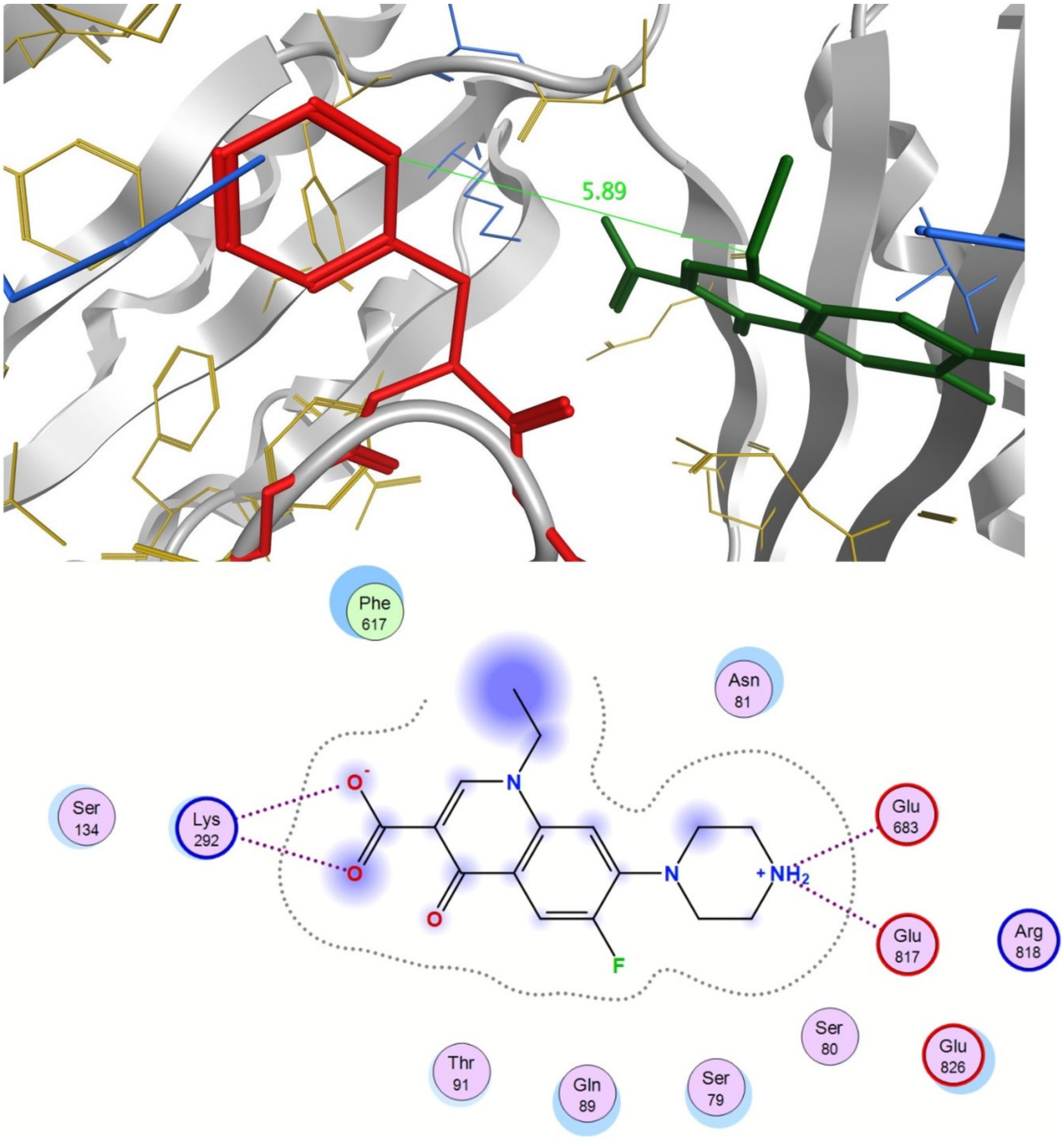

Figure S30. The most favourable pose of norfloxacin (green thick sticks) located in the proximal binding pocket (blue sticks) forms a hydrophobic interaction between C8 and the aromatic ring of Phe617.

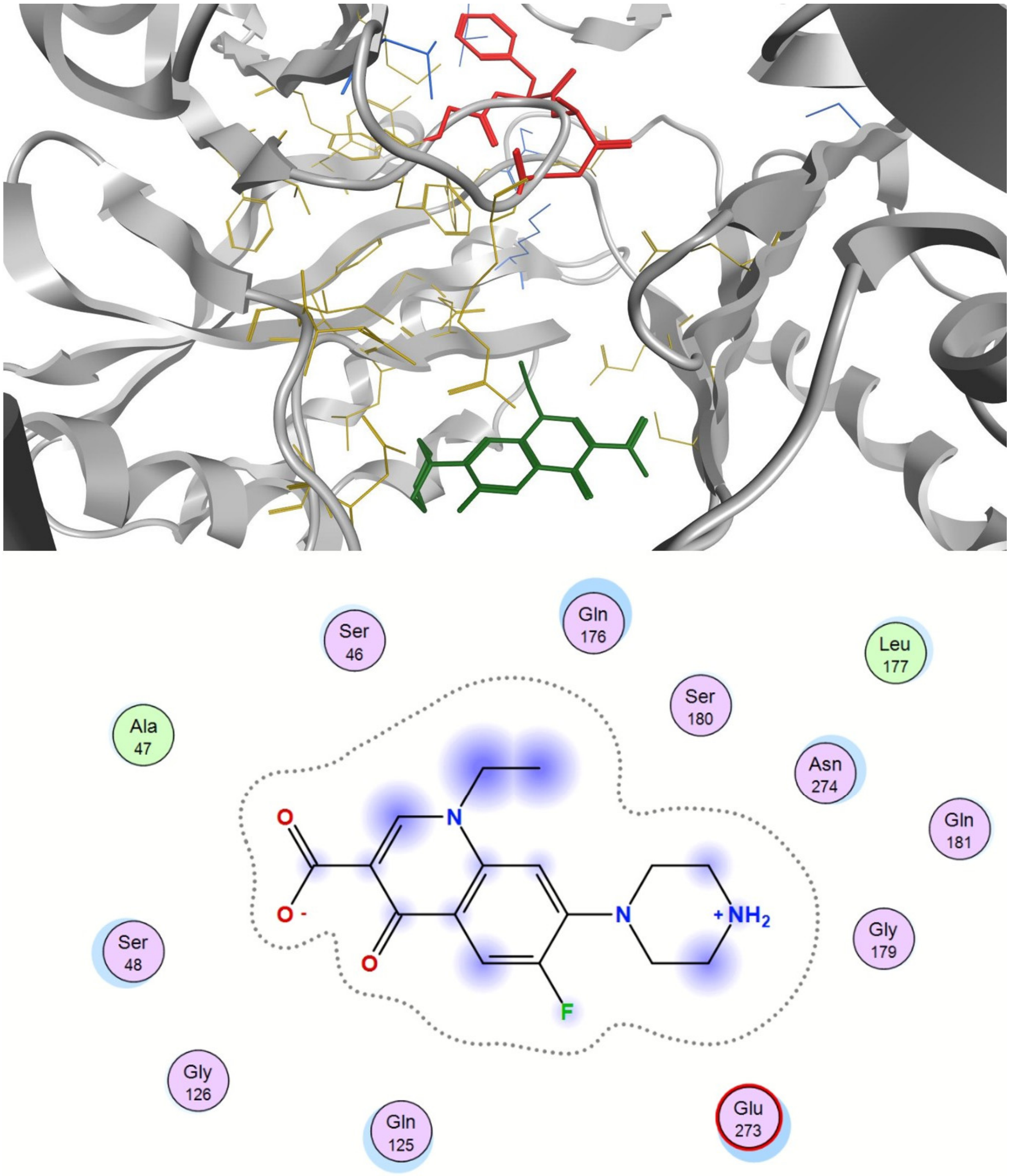

Figure S31. The second most favourable pose of norfloxacin (green thick sticks) located in the distal binding pocket (yellow sticks) established no interactions with the loop (red thick sticks).

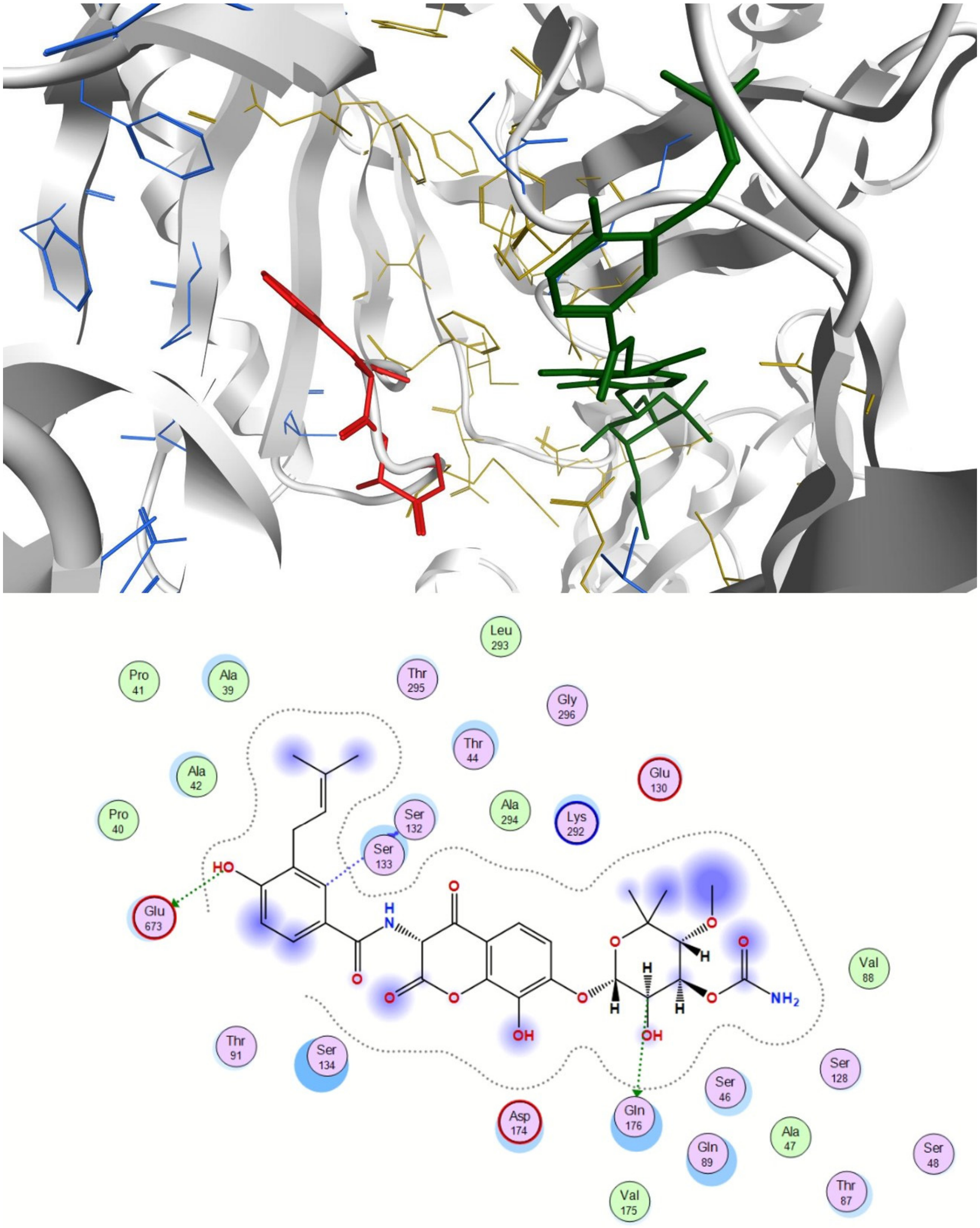

Figure S32. The most favourable pose of novobiocin (green thick sticks) located in the distal binding pocket (yellow sticks) does not interact with the loop (red thick sticks). The proximal binding pocket is shown in blue sticks.

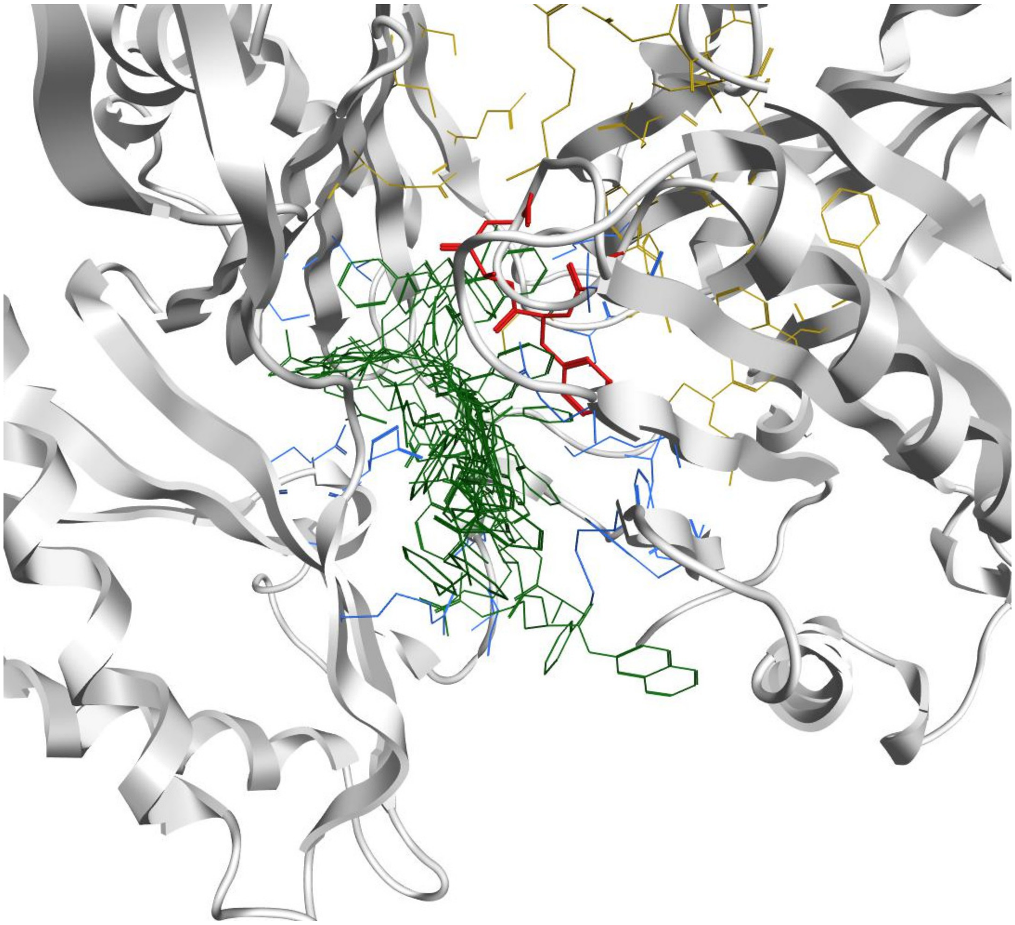

Figure S33. All 15 poses of PAβN (green stick representation) were found in the proximal binding pocket (blue sticks), with strong interactions with the glycine loop (red thick sticks) which regulates the passage of substrates between the proximal and distal pocket (yellow sticks). The distal binding pocket is shown in yellow sticks.

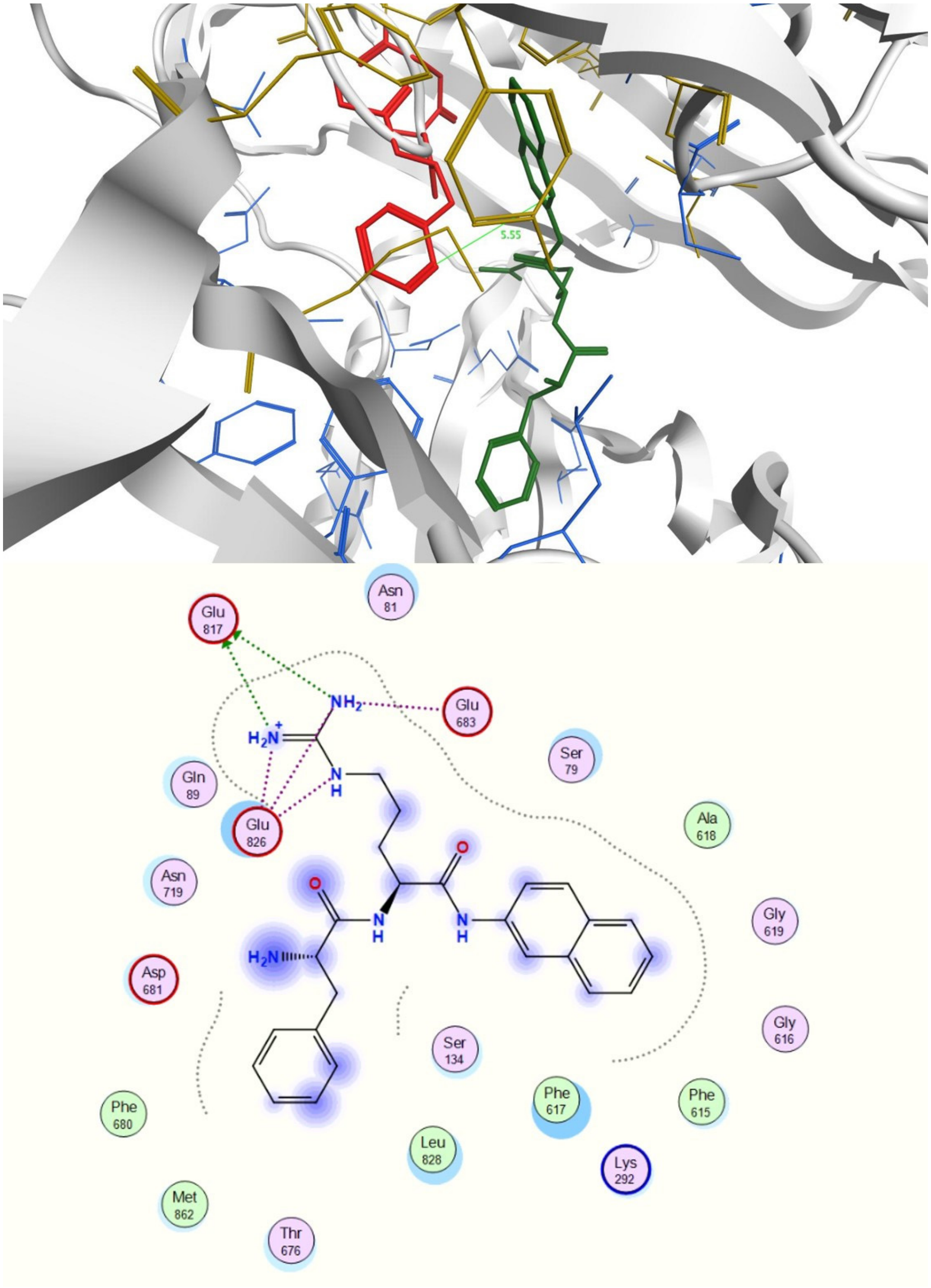

Figure S34. Most favourable pose of PAβN (green, thick lines representation) located in the periplasmic portion of the AcrB monomer of an E. Coli RND efflux pump (PDB ID 4DX5). An interaction can be seen with residues Phe617 and Ala618 which are part of the loop linking the distal and proximal binding pockets (pink, lines representation) and is thought to regulate passage of substrates through the pump.

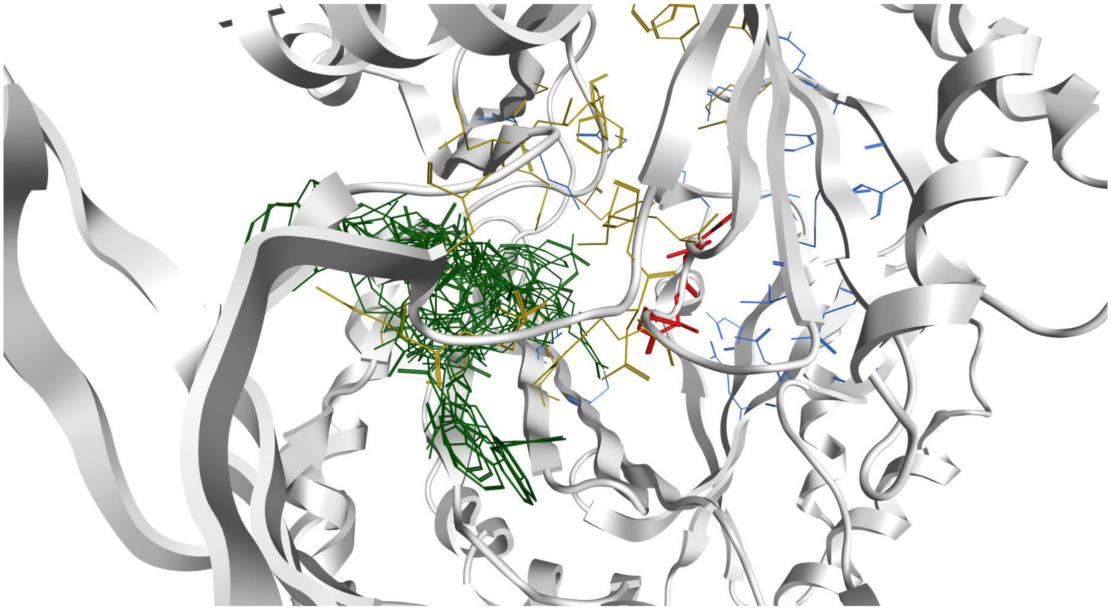

Figure S35. All poses of peptide **3** WRW (green sticks) located in the distal binding pocket (yellow sticks). The proximal binding pocket is shown as blue sticks and the loop as thick red sticks.

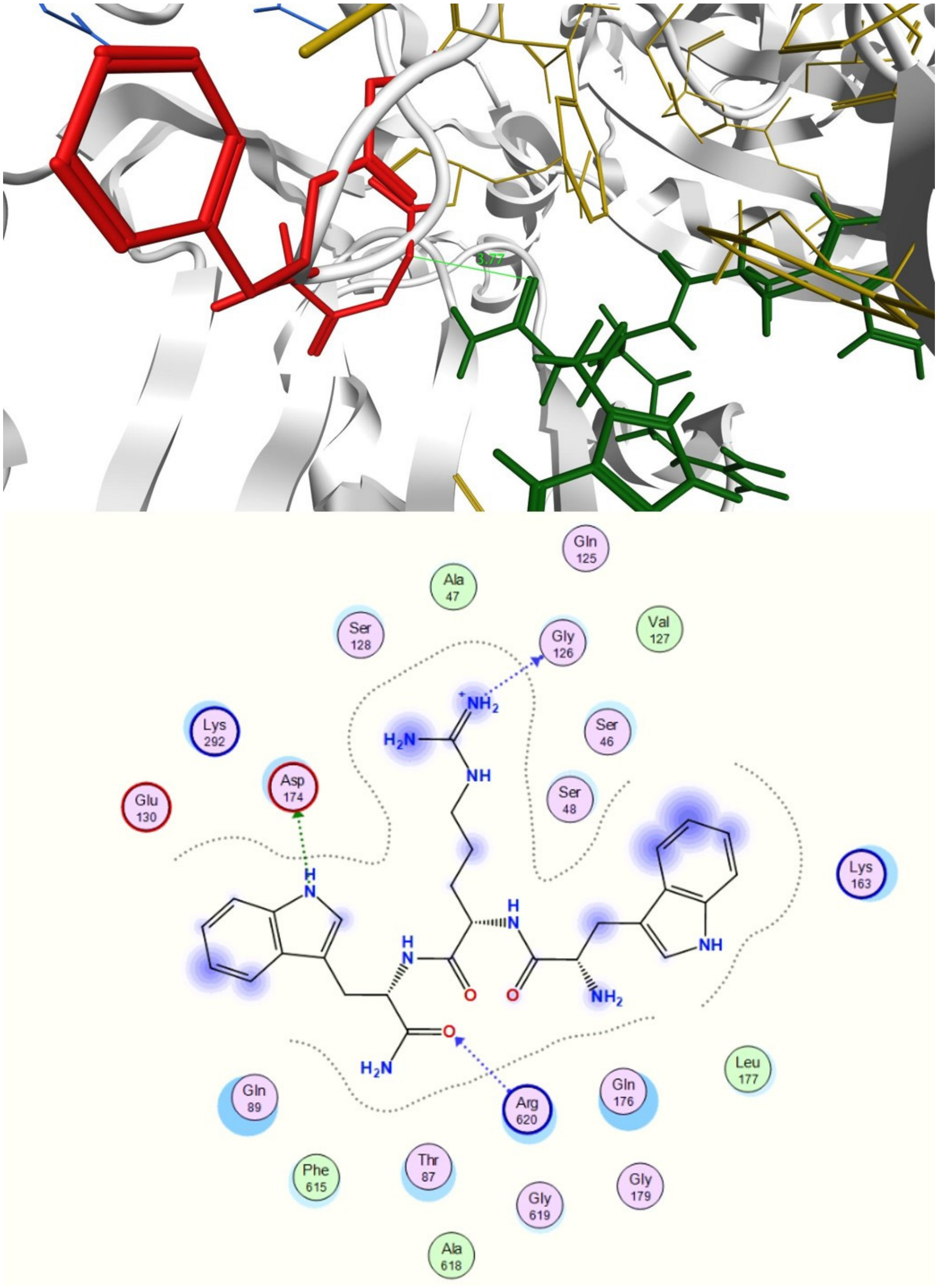

Figure S36. The most favourable pose of peptide **3** WRW (thick green sticks) located in the distal binding pocket (yellow sticks) shows an interaction with residues Ala618 and Gly619 of the loop (thick red sticks), but not with Phe617.

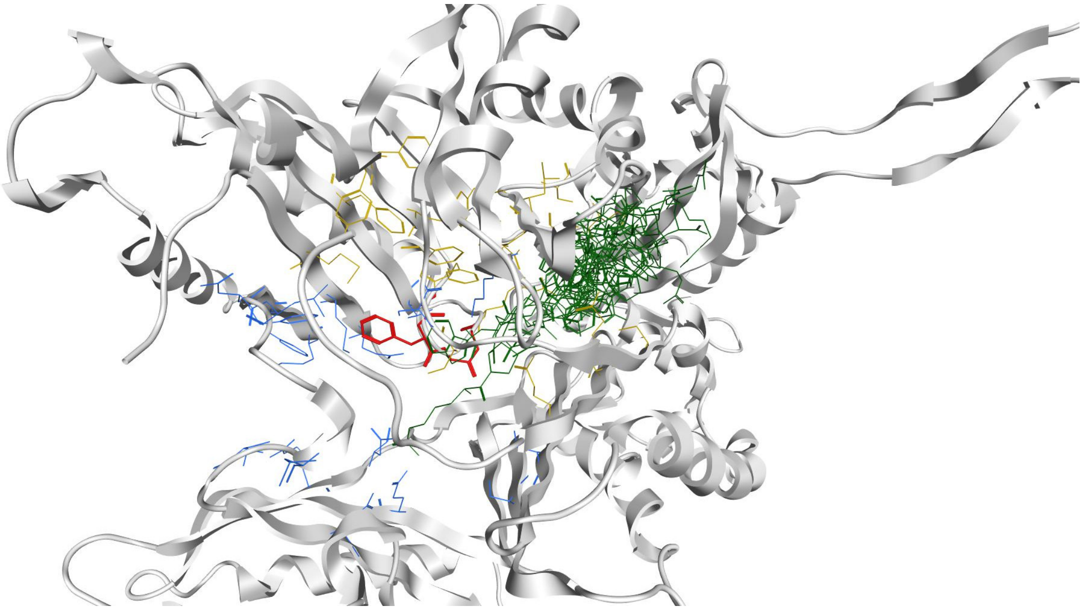

Figure S37. All poses of peptide **23** RPWPPR-NH_2 (green sticks), with the exception of the first two and the fourth, were found within the proximal distal binding pocket (yellow sticks) and no interactions were seen with the loop (thick red sticks).

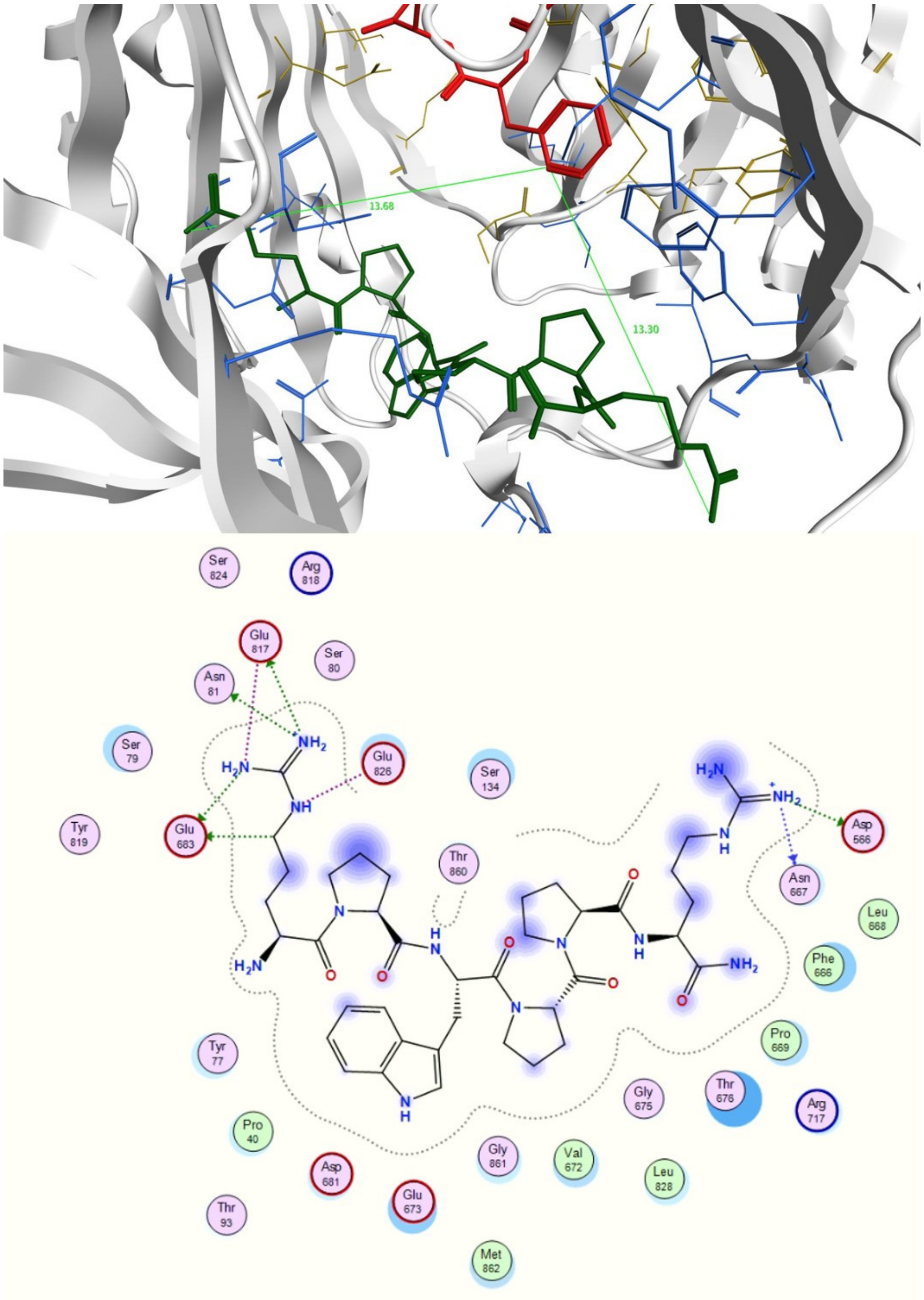

Figure S38. The most favourable pose of non potentiating peptide RPWPPR-NH2 (green sticks) located in the proximal pocket (blue sticks) does not form interactions with the loop (thick red sticks). The distal pocket is shown as yellow sticks.
